# Targeting Methionine Synthase in a Fungal Pathogen Causes a Metabolic Imbalance That Impacts Cell Energetics, Growth, and Virulence

**DOI:** 10.1128/mBio.01985-20

**Published:** 2020-10-13

**Authors:** Jennifer Scott, Monica Sueiro-Olivares, Benjamin P. Thornton, Rebecca A. Owens, Howbeer Muhamadali, Rachael Fortune-Grant, Darren Thomson, Riba Thomas, Katherine Hollywood, Sean Doyle, Royston Goodacre, Lydia Tabernero, Elaine Bignell, Jorge Amich

**Affiliations:** a Manchester Fungal Infection Group, Division of Infection, Immunity, and Respiratory Medicine, School of Biological Sciences, Faculty of Biology, Medicine and Health, University of Manchester, Manchester Academic Health Science Centre, Manchester, United Kingdom; b School of Biological Sciences, Faculty of Biology, Medicine and Health, University of Manchester, Manchester Academic Health Science Centre, Manchester, United Kingdom; c Department of Biology, Maynooth University, Maynouth, Co. Kildare, Ireland; d Department of Biochemistry, Institute of Integrative Biology, University of Liverpool, Liverpool, United Kingdom; e Manchester Institute of Biotechnology, University of Manchester, Manchester, United Kingdom; Universidade de Sao Paulo

**Keywords:** antifungal target, *Aspergillus fumigatus*, doxycycline, established infection, fungal virulence, methionine synthase, primary metabolism, target validation, TetOFF

## Abstract

There is an urgent need to develop novel antifungals to tackle the threat fungal pathogens pose to human health. Here, we have performed a comprehensive characterization and validation of the promising target methionine synthase (MetH). We show that in Aspergillus fumigatus the absence of this enzymatic activity triggers a metabolic imbalance that causes a reduction in intracellular ATP, which prevents fungal growth even in the presence of methionine. Interestingly, growth can be recovered in the presence of certain metabolites, which shows that *metH* is a conditionally essential gene and consequently should be targeted in established infections for a more comprehensive validation. Accordingly, we have validated the use of the tetOFF genetic model in fungal research and improved its performance *in vivo* to achieve initial validation of targets in models of established infection. We show that repression of *metH* in growing hyphae halts growth *in vitro*, which translates into a beneficial effect when targeting established infections using this model *in vivo*. Finally, a structure-based virtual screening of methionine synthases reveals key differences between the human and fungal structures and unravels features in the fungal enzyme that can guide the design of novel specific inhibitors. Therefore, methionine synthase is a valuable target for the development of new antifungals.

## Abstract (precis)

Fungal pathogens are responsible for millions of life-threatening infections on an annual basis worldwide. The current repertoire of antifungal drugs is very limited and, worryingly, resistance has emerged and already become a serious threat to our capacity to treat fungal diseases. The first step to develop new drugs is often to identify molecular targets in the pathogen whose inhibition during infection can prevent its growth. However, the current models are not suitable to validate targets in established infections. Here, we have characterized the promising antifungal target methionine synthase in great detail, using the prominent fungal pathogen Aspergillus fumigatus as a model. We have uncovered the underlying reason for its essentiality and confirmed its druggability. Furthermore, we have optimized the use of a genetic system to show a beneficial effect of targeting methionine synthase in established infections. Therefore, we believe that antifungal drugs to target methionine synthase should be pursued and additionally, we provide a model that permits gaining information about the validity of antifungal targets in established infections.

## INTRODUCTION

Fungal pathogens represent an increasing risk to human health (1), with over one billion people worldwide affected by mycoses annually. Many of these mycoses are superficial infections of the skin, nails or mucosal membranes and although troublesome are usually not life-threatening. However, some fungi cause devastating chronic and invasive fungal infections, which result in an estimated 1.6 million deaths per year (2). Incidences of invasive infections caused by *Aspergillus*, *Candida*, *Cryptococcus*, and *Pneumocystis* species are increasing (3), a cause for serious concern as these genera are responsible for 90% of deaths caused by mycoses (4). Despite the availability of antifungal drugs, the mortality rates for invasive aspergillosis, invasive candidiasis, cryptococcal meningitis, and Pneumocystis jirovecii pneumonia are intolerably high, reaching more than 80, 40, 50, and 30%, respectively (2, 5). There are currently only four classes of antifungals in clinical use to treat invasive infections (azoles, echinocandins, polyenes, and flucytosine), all suffering from pharmacological drawbacks, including toxicity, drug-drug interactions, and poor bioavailability (6, 7). With the sole exemption of flucytosine, which is only used in combinatory therapy with amphotericin B for cryptococcal meningitis and *Candida* endocarditis (7), the current antifungals target critical components of the fungal cell membrane or cell wall (8), which represents a very limited druggable space. The rise of antifungal resistance presents an additional challenge as mortality rates in patients with resistant isolates can reach 100%, making the development of new antifungal drugs increasingly critical for human health (1, 9). Targeting fungal primary metabolism is broadly considered a valid strategy for the development of novel antifungals, as it is crucial for pathogen virulence and survival (10, 11). A primary example of a success of this strategy is olorofim (F901318), a novel class of antifungal that targets the pyrimidine biosynthesis pathway (12), which is currently in clinical trials.

**FIG 1:**
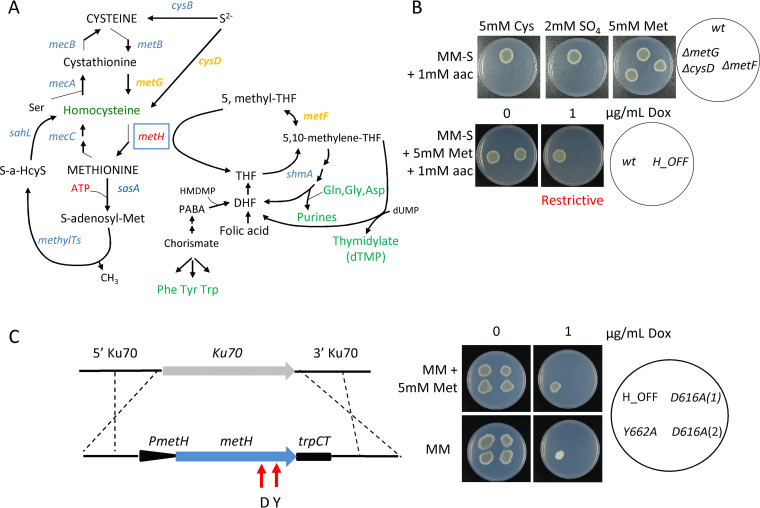
Methionine synthase (MetH) enzymatic activity is essential for A. fumigatus viability. (A) Schematic representation of the trans-sulfuration pathway and its intersection with the one carbon metabolic route. (B) Both strains, a *ΔmetF* mutant (which blocks the one carbon metabolism route) and a *ΔmetG ΔcysD* mutant (which blocks the trans-sulfuration pathway) could grow in the presence of methionine. In contrast, the *metH_tetOFF* strain (*H_OFF*) could not grow in restrictive conditions (+Dox) even if methionine was supplemented. The phenotypic analysis was repeated in three independent experiments. Representative plates are shown. (C) A second copy of the *metH* gene under the control of its own promoter was introduced in the innocuous *Ku70* locus of the *H_OFF* background strain. Two point-mutated versions of the gene were introduced, one that causes a D→A substitution in amino acid 616 and another one that causes a Y→A substitution in amino acid 662. In nonrestrictive conditions all strains were able to grow as the parental *H_OFF* strain. In the presence of Dox and absence of Met, the Y662A protein was able to trigger significant growth, suggesting that its enzymatic activity is impaired but not blocked. In the presence of Met, the Y662A strain grew as well under restrictive as under nonrestrictive conditions, suggesting that partial enzymatic activity is sufficient to cover the essential function of MetH. In restrictive conditions and absence of methionine, the D616A strain was not able to grow, indicating that enzymatic activity is blocked in this mutated protein. The D616A was also not able to grow in the presence of Dox and Met, indicating that enzymatic activity is required for the essential function of MetH. The phenotypic analysis was repeated in three independent experiments. Representative plates are shown.

Methionine synthases catalyze the transfer of a methyl group from *N*5-methyl-5,6,7,8-tetrahydrofolate (CH_3_-THF) to l-homocysteine (Hcy), as shown in Fig. 1A. Two unrelated protein families catalyze this reaction: cobalamin-dependent methionine synthases (EC 2.1.1.13) and cobalamin independent methionine synthases (EC 2.1.1.14). Members of both families must catalyze the transfer of a low active methyl group from the tertiary amine, CH_3_-THF, to a relatively weak nucleophile, Hcy sulfur. Cobalamin-dependent enzymes facilitate this transfer by using cobalamin as an intermediate methyl carrier (13). In contrast, cobalamin-independent enzymes directly transfer the methyl group from CH_3_-THF to Hcy (14). Logically, proteins of each family differ significantly both at the amino acid sequence (15) and three-dimensional (3D) structure level (16). Interestingly, fungi encode cobalamin-independent methionine synthases, whereas humans have only the cobalamin-dependent enzyme, opening the possibility to target fungal enzymes for antifungal therapy.

We have previously shown that the methionine synthase-encoding gene (*metH*, AFUA_4G07360) is essential for A. fumigatus viability and virulence, which led us to propose it as a promising target for antifungal drug development (17). In support of this, a systematic metabolic network analysis by Kaltdorf et al. identified methionine synthase as a promising antifungal drug target worthy of investigation (11). Methionine synthase has also been described as essential for Candida albicans viability (18, 19) and necessary for Cryptococcus neoformans pathogenicity (20), which suggests that a drug developed against this enzyme may have a broad spectrum of action. Moreover, fungal methionine synthases are cobalamin independent, differing significantly from the cobalamin-dependent human protein at the amino acid sequence level: only 11.2% identity, 20.4% similarity, and 60.2% gaps when the A. fumigatus and human proteins were aligned using L-Align from EMBL (21, 22). Therefore, it should be possible to develop drugs with low toxicity potential.

Target validation is critical and has been suggested as the most important step in translating a new potential target into a viable drug target because of its role in achieving efficacy in patients (23). Indeed, a retrospective analysis from AstraZeneca’s drug pipeline showed that projects that had performed a more thorough target validation were less likely to fail: 73% of the projects were active or successful in phase II compared to only 43% of projects without such extra target validation (24). Therefore, in the present study we aimed to further substantiate methionine synthase’s potential as an antifungal drug target, before advancing the drug discovery process. In particular, we were interested in (i) unraveling the mechanistic basis of methionine synthase essentiality in A. fumigatus, which is needed to fully explore the potential of this enzyme as a drug target and to be able to anticipate drug resistance mechanisms, and (ii) developing *in vivo* models of infection to mimic treatment against the target in an established infection and using them to validate methionine synthase as an antifungal drug target.

## RESULTS AND DISCUSSION

### Methionine synthase enzymatic activity is essential for *Aspergillus fumigatus* viability.

We had previously demonstrated that the methionine synthase encoding gene is essential for A. fumigatus viability and virulence (17); however, the underlying reason for this essentiality was still unclear. To address this question, we constructed strains that express the *metH* gene under the control of a tetOFF system recently adapted for *Aspergillus* (25) in two different A. fumigatus wild-type backgrounds, ATCC 46645 and A1160. The advantage of the tetOFF system over other regulatable systems is that doxycycline (Dox) can be added to downregulate gene expression in growing hyphae (see Fig. S1A in the supplemental material), and thus this system permits investigation of the consequences of the repression of an essential gene in growing mycelia. The constructed *metH_tetOFF* strains (*H_OFF*) grew as the wild type in the absence of Dox, but as little as 0.5 μg/ml was sufficient to completely prevent colony development on an agar plate even in the presence of methionine (see Fig. S1B). This corroborates our previous result that methionine synthase is essential for A. fumigatus viability, meaning that its absence does not simply cause methionine auxotrophy (17).

10.1128/mBio.01985-20.3 FIG S1 **(**A) The addition of 1 μg/ml Dox to a 16 h growing mycelia quickly downregulated transcription of *metH*. (B) The growth of two *metH_tetOFF* strains in two different backgrounds was prevented in the presence of as little as 0.5 μg/ml Dox, even in the presence of methionine. The phenotypic analysis was repeated in three independent experiments. Representative plates are shown. (C) The *ΔmetG ΔcysD* was able to grow on a medium supplemented only with Met, proving that it is a true methionine auxotroph. The auxotrophy could not be rescued with only 20 μM of methionine (the medium was prepared N-free to force Met uptake and supplemented with other amino acids to cover for nitrogen demand). (D) An A. fumigatus *ΔmetG ΔcysD* mutant (methionine auxotroph) was completely avirulent in a leukopenic murine model of invasive pulmonary aspergillosis. (E) A strain expressing a C-terminus-GFP-tagged MetH-D616 protein from the pJA49 overexpression plasmid (see Fig. S1F) in the *H_OFF* background strain showed fluorescence in –Dox conditions (did not grow in +Dox conditions, see Fig. S4A), proving that the point mutated MetH-D616 protein is stable. (F) Schematic representation of the pJA49 plasmid for episomal overexpression of genes in A. fumigatus. It carries the A. nidulans AMA1 autologous replicating sequence and the hygromycin B resistance gene (*hygrB*) as a selection marker. A unique StuI restriction site allows introduction of any PCR amplified ORF in-frame under the control of the A. fumigatus strong promoter *hspA* and the A. nidulans *trpC* terminator. (G) Schematic representation of the genes overexpressed (in green) to eliminate potential accumulation of toxic homocysteine and derivatives (in red). (H) Fold change in expression level of *mecA* and *sahL* measured by RT-PCR. Both genes are highly overexpressed from plasmid pJA49. (I) Expression level of *blhA* determined by retrotranscription and PCR (the fold change cannot be calculated since this gene is not expressed in A. fumigatus). Download FIG S1, TIF file, 2.6 MB. Copyright © 2020 Scott et al. 2020 Scott et al. This content is distributed under the terms of the Creative Commons Attribution 4.0 International license.

Methionine synthase forms an intersection between the trans-sulfuration pathway and the one carbon metabolic route (Fig. 1A), as the enzyme utilizes 5-methyl-tetrahydrofolate as cosubstrate. Therefore, *metH* might be essential due to the disruption of the trans-sulfuration pathway or of the one carbon metabolic route upon its absence. Alternatively, it could be that the presence of the enzyme itself is essential, either because its enzymatic activity is required or because it is fulfilling an unrelated additional role, such as being part of a multiprotein complex. To differentiate between these possibilities, we constructed a double *ΔmetG ΔcysD* mutant, blocked in the previous step of the trans-sulfuration pathway, and a *ΔmetF* deletant, which blocks the previous step of the one-carbon metabolic route (Fig. 1A). As we had previously observed (26), to fully rescue *ΔmetF*’s growth the media had to be supplemented with methionine and other amino acids (see Fig. S1C), as the folate cycle is necessary for the interconversion of serine and glycine and plays a role in histidine and aromatic amino acid metabolism (27, 28). Consequently, we added a mix of all amino acids except cysteine and methionine (aac) to the S-free medium for this experiment. Phenotypic tests (Fig. 1B) confirmed that the *ΔmetG ΔcysD* and *ΔmetF* mutants were viable and could grow in the presence of methionine. In contrast, the *H_OFF* conditional strain could not grow under restrictive conditions even in the presence of the amino acid mix and methionine (Fig. 1B). Therefore, the MetH protein itself, and not the integrity of the trans-sulfuration and one-carbon pathways, is essential for A. fumigatus viability. Interestingly, the true methionine auxotroph *ΔmetG ΔcysD* (see Fig. S1C) was avirulent in a leukopenic model of pulmonary aspergillosis (see Fig. S1D), suggesting that the amount of readily available methionine in the lung is very limited, not sufficient to rescue its auxotrophy. Indeed, the level of methionine in human serum was calculated to be as low as ∼20 μM (29, 30), which was described as insufficient to support the growth of various auxotrophic bacterial pathogens (31), and we have also observed that this is not enough to rescue growth of the A. fumigatus *ΔmetG ΔcysD* auxotroph (Fig. S1C).

The essentiality of the MetH protein could be directly linked to its enzymatic activity or, alternatively, the protein could be performing an additional independent function. To discern between these two possibilities, we constructed two strains that express single-point mutated versions of MetH from the neutral Ku70 locus (32) of the *H_OFF* background strain, under the control of its native promoter (Fig. 1C). These point mutations, *metH^g2042A>C^* (D616A) and *metH^g2179TA>GC^* (Y662A), were previously described to prevent conformational rearrangements required for activity of the C. albicans methionine synthase (33). In the absence of Dox, these strains grew normally, as they expressed both the wild-type MetH, from the tetOFF promoter, and the mutated version of the protein (Fig. 1C). In the presence of Dox, when the wild-type *metH* gene was downregulated, the Y662A strain grew on sulfate worse than in nonrestrictive conditions, but still to a significant extent, suggesting that this point mutation did not completely abrogate enzymatic activity (Fig. 1C). Interestingly, Y662 grew normally on methionine, showing that methionine can compensate for a partial reduction of MetH activity (Fig. 1C). The D616A mutated protein was confirmed to be stable as a green fluorescent protein (GFP)-tagged version of this protein could be visualized under nonrestrictive (–Dox) conditions (see Fig. S1E and strain discussed in more detail later in this article). Interestingly, the D616A strain (two isolates were tested) was not able to grow on sulfate (Fig. 1C), demonstrating that enzymatic activity was fully blocked. Nor could it grow on methionine (Fig. 1C), indicating that enzymatic activity is required for viability even in the presence of the full protein. All of these phenotypes support the conclusion that methionine synthase enzymatic activity is required for viability.

### Absence of methionine synthase enzymatic activity results in a shortage of crucial metabolites but does not cause toxic accumulation of homocysteine.

**FIG 2:**
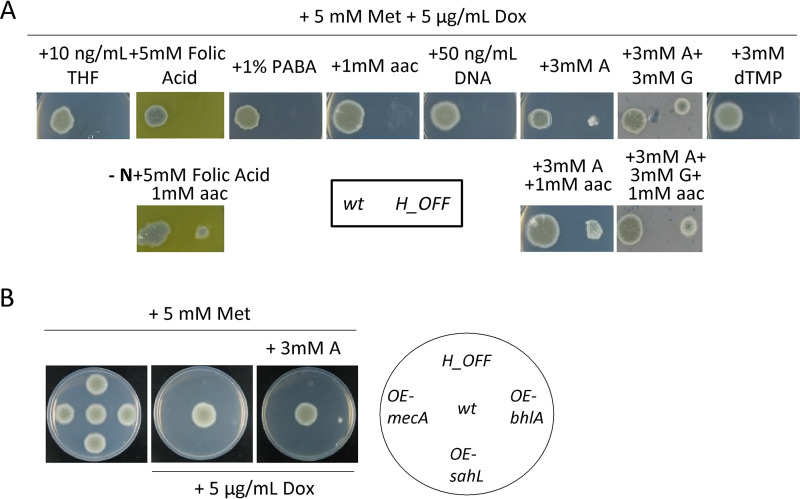
Shortage of important downstream metabolites, but not the toxic accumulation of homocysteine, partially accounts for MetH essentiality. (A) Minimal medium containing 5 mM Met and 5 μg/ml Dox was supplemented with a variety of metabolites (potentially relevant for downstream processes [see Fig. 1A]) in single and multiple addition. Growth assays showed that purines (adenine A and guanine G, 3 mM) could slightly reconstitute growth of the *H_OFF* strain. Additional supplementation with amino acids improved growth, but not to the wild-type levels. Folic acid (5 mM) could also reconstitute growth, but only when amino acids were the only N source. (B) Overexpression of genes that could detoxify a potential accumulation of homocysteine did not reconstitute growth in the absence of MetH activity. Further addition of adenine did not improve growth. The phenotypic analyses were repeated in three independent experiments. Representative plates are shown.

The absence of methionine synthase enzymatic activity has two direct consequences, which could cause deleterious effects and therefore explain its essentiality even under methionine supplementation (Fig. 1A). It could cause an accumulation of the potentially toxic substrate homocysteine and/or a shortage of the coproduct tetrahydrofolate (THF). THF is directly converted to 5,10-methylene-THF, which is required for the synthesis of purines and thymidylate and thus for DNA synthesis; additionally, since purine biosynthesis requires Gln, Gly, and Asp, and as THF *de novo* synthesis requires chorismate (precursor of aromatic amino acids), a shortage of THF might cause a depletion of amino acids (Fig. 1A). To investigate whether the depletion of any of these metabolites underlies MetH essentiality, we supplemented the media with a number of precursors and potentially depleted metabolites (Fig. 2A). Added as the sole supplement, only adenine was able to trigger growth, but to a minimal degree. Further addition of a mixture of all amino acids noticeably improved growth. Supplementation with adenine and guanine (purine bases) did also reconstitute noticeable growth, which was not enhanced with further addition of amino acids. Folic acid was also capable of reconstituting growth, but only when amino acids were added as the sole N source (Fig. S2A). However, no combination of compounds was able to reconstitute growth to the wild-type level. This suggests that a shortage of relevant metabolites derived from THF, predominantly adenine, partially accounts for methionine synthase essentiality even under methionine supplementation but cannot explain it completely. In other fungi, such as Pichia pastoris (34) or Schizosaccharomyces pombe (35), supplementation with methionine and adenine was found to restore growth of a *metH* mutant to wild-type levels, indicating that these are combined auxotrophs. In A. fumigatus it seems to be more complex because supplementation with methionine, adenine, and other amino acids could still not fully restore growth, suggesting that more factors are implicated.

10.1128/mBio.01985-20.4 FIG S2 **(**A) Relevance of the nitrogen source for the capacity of different metabolites to reconstitute growth of the *H_OFF* strain in restrictive conditions. Amino acids alone were not able to reconstitute growth. Folic acid could partially and ATP and SAM could completely reconstitute growth when amino acids (aac) were the only N source, suggesting that they are only taken up when there is a high variety of permeases in the cell membrane. Pyruvate could reconstitute growth to the wild-type level in the absence of glucose and independently of the in presence of amino acids, although growth improved when they were added. (B) Supplementation with ATP and AMP reconstituted growth (when amino acids were the only N source) of the *H_OFF* strain in restrictive conditions. Download FIG S2, TIF file, 2.5 MB. Copyright © 2020 Scott et al. 2020 Scott et al. This content is distributed under the terms of the Creative Commons Attribution 4.0 International license.

To investigate whether homocysteine could be accumulating to toxic levels in the absence of MetH activity, we overexpressed several genes that should alleviate its accumulation. To do this, we designed and constructed the plasmid pJA49, which allows direct integration of any open reading frame (ORF) to episomally overexpress genes in A. fumigatus. Plasmid pJA49 carries the A. nidulans AMA1 autologous replicating sequence (36, 37) and the hygromycin B resistance gene (*hygrB*) as a selection marker. A unique StuI restriction site allows introduction of any PCR amplified ORF in frame under the control of the A. fumigatus strong promoter *hspA* (38) and the A. nidulans *trpC* terminator (see Fig. S1F). Using this plasmid, we produced a strain in the *H_OFF* background that episomally overexpresses *mecA*, encoding cystathionine-β-synthase, which converts homocysteine to cystathionine (Fig. 1A). Homocysteine exerts toxic effects through its conversion to *S*-adenosylhomocysteine, which causes DNA hypomethylation (39, 40), or to homocysteine thiolactone, which causes *N*-homocysteinylation at the ε-amino group of protein lysine residues (41, 42). Consequently, we also constructed strains that episomally overexpress genes that could detoxify those products: the *S*-adenosyl-homocysteinase lyase encoding gene *sahL* (AFUA_1G10130) or the A. nidulans homocysteine thiolactone hydrolase encoding gene *blhA* (AN6399) (A. fumigatus genome does not encode any orthologue) (see Fig. S1G). However, despite a strong overexpression of the genes (see Fig. S1H and I), none of them could rescue growth of the *H_OFF* strain in restrictive conditions (Fig. 2B). Therefore, our overexpression experiments suggest that homocysteine accumulation is not responsible for *metH* essentiality, but additional experiments such as quantification of homocysteine levels, which are currently challenging, would be required to further support this hypothesis. The addition of adenine to the medium did not improve growth of the overexpression strains further than that of the *H_OFF* background (Fig. 2B), indicating that methionine synthase essentiality seemingly is not a combined effect of homocysteine accumulation and depletion of THF-derived metabolites. Toxic accumulation of homocysteine was speculated to be the underlying reason of methionine synthase essentiality in both Candida albicans and Cryptococcus neoformans (19, 20), but our results suggest that this is not the case in A. fumigatus. Therefore, we propose that the previous assumption should be revisited in other fungal pathogens.

### Methionine synthase repression triggers a metabolic imbalance that causes a decrease in cell energetics.

**FIG 3:**
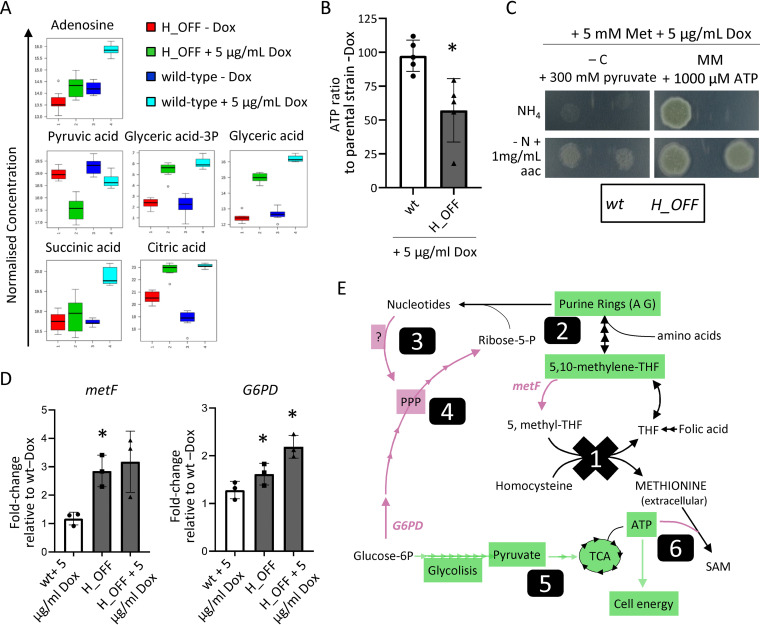
Lack of methionine synthase activity causes a decrease in cell energetics. (A) Normalized concentrations of metabolites in fungal mycelia (*n *=* *8). Adenosine levels were decreased in *H_OFF*+Dox compared to wild type + Dox (wt+Dox), which agrees with its capacity to partially reconstitute growth. Several metabolites of the glycolysis and TCA pathways were reduced in *H_OFF*+Dox compared to wt+Dox, suggesting low energetic levels. (B) The levels of ATP significantly decreased in the *H_OFF* strain upon Dox addition, while they did not vary significantly in the wild-type strain. Each point represents a biological replicate, which was assayed with three technical replicates. Data were analyzed by using a one-sample *t* test to a hypothetical value of 100 (i.e., no change in ratio of ATP). Graph displays the mean and standard deviations (SD). (C) Pyruvate as the sole carbon source could reconstitute growth of the *H_OFF* strain in restrictive conditions to wild-type levels. For both strains, growth was limited and slightly improved in the presence of amino acids (aac). ATP could fully reconstitute growth of *H_OFF* when amino acids were the sole N source. The phenotypic analyses were repeated in three independent experiments. Representative plates are shown. (D) RT-PCR calculation of the fold change in genetic expression of *metF* and *G6PD* with respect to their expression in wild type without Dox. Each point represents a biological replicate which was analyzed with three technical replicates. Data were analyzed by using a one-sample *t* test to a hypothetical value of 1 (i.e., no change in expression). The graphs display the means and SD. (E) Schematic representation of the metabolic imbalance started with MetH repression (1). Lack of the enzymatic activity caused a shortage of 5,10-methylene-THF and consequently of purine rings (2). This was sensed as a shortage of nucleotides that the cell attempted to compensate through an unknown mechanism, seemingly TOR and PKA independent (3), that activated glucose flow through the pentose phosphate pathway (PPP) (4). That caused a reduction of glycolysis and TCA cycles, which in turn decreased ATP levels (5). ATP usage for *S*-adenosylmethionine (SAM) synthesis was maintained, which caused a drop in cell energy that resulted in growth arrest (6). Genes/pathways/compounds expected to be reduced are highlighted in green and those increased in magenta.

Aiming to identify any adverse metabolic shift in the absence of MetH and/or accumulation of toxic compounds that could explain its necessity for proper growth, we performed a metabolomics analysis, via gas chromatography-mass spectrometry (GC-MS), comparing the metabolites present in wild-type and *H_OFF* strains before and 6 h after Dox addition. Before Dox addition, both strains clustered closely together in a principal component analysis (PCA) scores plot (see Fig. S3A), showing that their metabolic profiles are highly similar. However, 6 h after Dox addition the strains clusters became clearly separated, denoting differential metabolite content. Analysis of the differentially accumulated metabolites (a full list is shown in Table S1 in the supplemental material) using the online platforms MBRole (43) and Metaboanalyst (44, 45) did not reveal any obvious metabolic switch, probably due to the rather small number of metabolites that could be identified by cross-referencing with the Golm library (http://gmd.mpimp-golm.mpg.de/). Manual inspection of the metabolites pointed out interesting aspects. First, the methionine levels were not significantly different, which demonstrates that methionine supplementation in the growth medium triggers correct intracellular levels in the *H_OFF* strain; this undoubtedly rules out that a shortage of methionine could be the cause of the essentiality of methionine synthase. Second, we detected a significantly smaller amount of adenosine in the *H_OFF* strain compared to the wild-type after Dox addition (Fig. 3A), which is in agreement with our previous result that supplementation of adenine can partially reconstitute growth in the absence of MetH. We did not find accumulation of compounds with a clear toxic potential upon *metH* repression. Nevertheless, we detected a smaller amount of several amino acids (Phe, Ser, Glu, Pro, Ile, Thr, Ala, and Asp; see Fig. S3B), which may suggest that the cells have a reduced growth rate upon *metH* repression. Interestingly, we noticed a significantly lower accumulation of some metabolites of the glycolysis pathway and tricarboxylic acid (TCA) cycle (Fig. 3A) and some other mono- and polysaccharides (see Fig. S3B). These variations could reflect a low energetic status of the cells upon *metH* repression. Indeed, we found that the level of ATP significantly decreased in the *H_OFF* strain, but not in the wild type, upon Dox addition (Fig. 3B). Therefore, we evaluated whether supplementation of the medium with substrates that have the potential to increase cell energetics can rescue *H_OFF* growth in restrictive conditions. We found that when pyruvate, which can directly be converted to acetyl coenzyme A to enter the TCA cycle, was added as the sole carbon source *H_OFF* growth was reconstituted in restrictive conditions to the same level as the wild type (Fig. 3C). Growth was limited for both strains, since pyruvate does not appear to be a good carbon source (46). However, the presence of glucose in the medium precluded the reconstitution of growth of *H_OFF* (see Fig. S2A), probably because glucose prevents pyruvate uptake, as has been been reported in S. cerevisiae (47). We next tested the capacity of ATP to be used as an alternative energy source and to reconstitute growth. To diversify the presence of permeases in the cell membrane, and thus maximize the chance of ATP uptake, we assayed two different N sources: ammonium (NH_4_^+^, preferred source) and amino acids (see Fig. S2A). Indeed, when amino acids were the only N source, supplementation of the medium with ATP reconstituted *H_OFF* growth in restrictive conditions to wild-type levels (Fig. 3C; see also Fig. S2A and B in the supplemental material). This agrees with the recent observation that eukaryotic cells can uptake ATP and exploit it as an energy source (48). Interestingly, AMP supplementation also reconstituted *H_OFF* growth in restrictive conditions to wild-type levels (Fig. S2B). We speculate that this might be due to the known functions of AMP to activate AMP-activated protein kinase (AMPK; an energy sensor that restores cellular energy balance by switching on catabolic, ATP-generating pathways while switching off anabolic, ATP-consuming pathways [49]) and to stimulate glycolysis (via activation of phosphofructokinase [50, 51]). In conclusion, a decrease in cell energetics developed in the absence of methionine synthase seems to explain MetH essentiality for growth.

10.1128/mBio.01985-20.5 FIG S3 (A) Principal component analysis (PCA) scores plot of the GC-MS metabolome analysis. The wild-type and *H_OFF* strains clusters were close before addition of Dox and became clearly separated upon 6 h incubation in the presence of Dox. (B) The content of several amino acids and sugars were reduced in the H_OFF+Dox sample compared to the developmentally matched wt+Dox sample. (C) Neither low concentrations of rapamycin (to partially inhibit TOR) nor of H89 (to partially inhibit PKA) were able to reconstitute growth of the *H_OFF* strain in restrictive conditions. This suggests that these regulatory pathways are not responsible for the deleterious switch in metabolism that causes a drop in cellular ATP. Download FIG S3, TIF file, 1.2 MB. Copyright © 2020 Scott et al. 2020 Scott et al. This content is distributed under the terms of the Creative Commons Attribution 4.0 International license.

10.1128/mBio.01985-20.10 TABLE S1 Differentially accumulated metabolites detected in the metabolome analysis and identified using the Golm library. Download Table S1, PDF file, 0.5 MB. Copyright © 2020 Scott et al. 2020 Scott et al. This content is distributed under the terms of the Creative Commons Attribution 4.0 International license.

The fact that growth in the absence of methionine synthase can be reconstituted when there are sufficient levels of methionine and ATP implies that *metH* is a conditionally essential gene, meaning that it is only essential in the absence of the specific conditions that overcome the disturbances derived from its deficiency. We believe that a significant number of genes previously described as essential in fungi would in fact be conditionally essential; however, the right conditions to reconstitute growth have not been identified in many cases. This highlights a paramount consideration for the proper identification and validation of drug targets: the deficiencies introduced by targeting a conditionally essential gene must not be overcome during infection. This important concept has already been discussed by others (52–55), and we believe addressing it should become a priority for proper validation of antimicrobial targets. In the case of methionine synthase, it is unlikely that the fungus could acquire sufficient levels of ATP (combined with methionine and not using a preferred N source) in the lung tissue to overcome the growth defect resulting from targeting MetH. The concentration of free extracellular ATP in human plasma has been calculated to be in the submicromolar range (28 to 64 nM) (56). In the lungs, extracellular ATP concentrations must be strictly balanced and increased levels are implicated in the pathophysiology of inflammatory diseases (57); nevertheless, even in such cases ATP levels have been calculated in the low micromolar range (58, 59). Despite this low concentration and consequently unlikely compensation, we believe that MetH needs to be validated in a suitable model to confirm that its deficiency cannot be not overcome in established infections.

We then questioned how the lack of methionine synthase’s enzymatic activity could cause a drop in cell energy. We hypothesized that blockage of methionine synthase activity likely causes a forced conversion of 5,10-methylene-THF to 5-methyl-THF by the action of MetF (Fig. 1A). In support of this, we observed that expression of *metF* was increased in the *H_OFF* strain (Fig. 3D). This likely causes a shortage of 5,10-methylene-THF, since the conversion is not reversible and THF cannot be recycled by the action of methionine synthase (Fig. 1A). Indeed, supplementation of folic acid (only when amino acids are the sole N source; see Fig. S2A in the supplemental material) and of purine bases could partially restore growth (Fig. 2A), since they compensate for the deficit in purine ring biosynthesis when there is a shortage of 5,10-methylene-THF. However, this still does not explain why there is a drop in ATP. We hypothesized that the block of purine biosynthesis might be sensed as a shortage of nucleotides. This could then cause a shift in glucose metabolism from glycolysis and the TCA cycle (which produce energy) to the pentose phosphate pathway (PPP), which is required to produce ribose-5-phosphate, an integral part of nucleotides. In support of our hypothesis, a recent publication found that a feedback-insensitive, constitutively active methylene-tetrahydrofolate reductase (=MetF) enzyme causes depletion of nucleotides and reduction of ATP in S. cerevisiae (113). It has also been described that activation of anabolism in S. cerevisiae implies increased nucleotide biosynthesis and consequently metabolic flow through the PPP (60). To evaluate our hypothesis, we investigated the transcription level of the glucose-6-phosphate dehydrogenase (G6PD) encoding gene (AFUA_3G08470), which catalyzes the first committed step of the PPP. In agreement with our hypothesis, the expression of G6PD encoding gene increases in the *H_OFF* strain upon addition of Dox (Fig. 3D), likely reflecting an increased flow through the PPP. We then wondered how cells may be activating the PPP. The target of rapamycin (TOR) TORC1 effector, which is widely known to activate anabolism and growth (61–63), has been described to activate the PPP in mammalian cells (64, 65) and has been functionally connected with energy production and nucleotide metabolism in A. fumigatus (66). In addition, the cAMP/PKA (cyclic AMP/protein kinase A) pathway is known to be paramount for sensing of nutrients and the correspondent adaptation of gene expression and metabolism (67) and was found to be implicated in the regulation of nucleotide biosynthesis in A. fumigatus (68). Consequently, we explored whether a partial block of TOR with low concentrations of rapamycin or of PKA with H-89 could prevent the imbalanced activation of the PPP in the absence of MetH activity. However, neither of the inhibitors could reconstitute growth of the *H_OFF* strain in restrictive conditions (see Fig. S3C). This means that neither the TOR nor the PKA pathways seem to be involved in the deleterious metabolic shift that seemingly activates PPP and decreases flux through glycolysis. Therefore, more experiments are required to elucidate the mechanism underlying the metabolic imbalance developed upon *metH* downregulation.

In summary, we propose that absence of methionine synthase activity causes a strong defect in purine biosynthesis that the cell tries to compensate for by shifting carbon metabolism to the PPP; this metabolic imbalance causes a drop of ATP levels, which collapses cell energetics and results in halted growth (Fig. 3E).

Interestingly, we also detected that *metF* expression is higher in the *H_OFF* strain compared to the wild-type, even in the absence of Dox (Fig. 3D). This could be explained as an effort to compensate a higher demand of 5-methyl-THF by the slightly increased amount of methionine synthase in this strain (Fig. S1A). This effect could cause a mild defect in purine biosynthesis in the *H_OFF* strain, and indeed adenosine content was lower in the *H_OFF*–Dox condition compared with the wild-type–Dox sample in the metabolome analysis (Fig. 3A). Furthermore, this also explains why we detected a small but significant increase of G6PD expression in the *H_OFF*–Dox condition (Fig. 3D). Therefore, it seems that upregulating methionine synthase has the potential to cause the same metabolic imbalance as downregulating it. However, the effect of overexpression (notice that it is only ∼1.5-fold in our strain; see Fig. S1A) is minor and does not have obvious consequences for growth, since THF can be recycled, and thus the shortage of 5,10-methylene-THF is not severe. In any case, two important points can be highlighted from this small imbalance. First, methionine synthase activity is very important and must be finely tuned to maintain a proper metabolic homeostasis. Second, changing the expression level of genes with constitutive and/or regulatable promoters can have unexpected and hidden consequences that often go unnoticed.

### Supplementation with *S*-adenosylmethionine reconstitutes ATP levels and growth.

**FIG 4:**
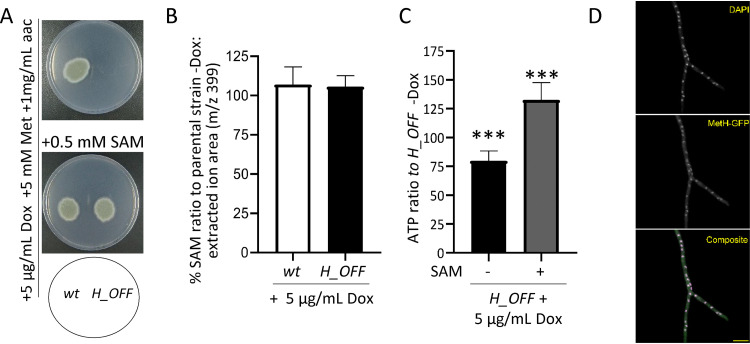
External SAM reconstitutes ATP levels and growth. (A) The addition of SAM to the medium reconstituted growth of the *H_OFF* strain in restrictive conditions. The phenotypic analysis was repeated in two independent experiments. Representative plates are shown. (B) The levels of SAM did not significantly decrease upon Dox administration (5 μg/ml) in wild-type or *H_OFF* strains. Graphs depict the means and SD of three biological and two technical replicates. Data were analyzed by using one-way analysis of variance with a Bonferroni posttest adjustment. (C) The presence of SAM in the medium (0.5 mM) prevented the decrease in ATP levels observed in the *H_OFF* strain upon Dox addition. In fact, ATP levels were increased compared to the minus Dox condition. Graphs depict the means and SD of two biological and four technical replicates. (D) Expression of MetH-GFP in the *H_OFF* background showed that the protein localized in both the cytoplasm and the nucleus. Singular channels are shown in gray scale. In the composite image, magenta indicates DAPI and green indicates GFP. Scale bar, 10 μm.

We have shown that the absence of MetH activity causes a reduction in ATP levels. *S*-Adenosylmethionine (SAM) is produced from methionine and ATP by the action of *S*-adenosylmethionine synthetase SasA (Fig. 1A), an essential enzyme in A. nidulans (69). Hence, we reasoned that the absence of MetH activity might cause a decrease in SAM levels. To test that hypothesis, we first attempted to rescue growth of the *H_OFF* strain in a medium supplemented with methionine and SAM. We tested various N sources to diversify the presence of permeases in the cell membrane, aiming to maximize the chances of SAM uptake (see Fig. S2A). Indeed, the addition of SAM reconstituted growth of the *H_OFF* strain in restrictive conditions in the presence of methionine when amino acids were the only N-source (Fig. 4A; see also Fig. S2 in the supplemental material). We then measured the intracellular concentration of SAM in growing mycelia upon addition of Dox using tandem mass spectrometry (MS/MS). Surprisingly, we observed that addition of Dox to the *H_OFF* strain did not cause a significant reduction in SAM levels (Fig. 4B). Consequently, we wondered how the addition of SAM may reconstitute *H_OFF* growth if its levels are not reduced upon *metH* repression. We speculated that as SAM is a crucial molecule it continues to be produced even if the levels of ATP are reduced, draining it from other cellular processes and thus triggering energy deprivation. In support of this hypothesis, we observed that supplementing SAM to the medium increased the levels of ATP in growing hyphae (Fig. 4C). Similarly, futile cycles of SAM formation and recycling were shown to cause ATP depletion upon deregulation of methylenetetrahydrofolate reductase (=MetF) activity in S. cerevisiae (113). It is also possible that added SAM allosterically inhibits MetF activity (113), preventing development of the deleterious metabolic imbalance, but this is unlikely as the levels of SAM did not decrease upon Dox addition in the *H_OFF* strain.

Since SAM supplementation can reconstitute *H_OFF* growth, it constitutes another condition that overcomes the conditional essentiality of *metH*, highlighting again the need to validate MetH in a model of established infection. The concentration of SAM in human serum is extremely low, in the range of 100 to 150 nM (30), and consequently it is unlikely that the fungus could find sufficient SAM during infection to compensate for the defect in ATP caused by targeting methionine synthase.

*S*-Adenosylmethionine plays a fundamental role as methyl donor for the majority of cellular methylation reactions, including methylation of DNA. Given the observed importance of SAM in the absence of MetH activity and considering that in P. pastoris and C. albicans methionine synthase was reported to localize in the nucleus, as well as in the cytoplasm (34), we speculated that nuclear localization might be important for MetH cellular function. To test this hypothesis, we constructed strains expressing different versions of C terminus GFP-tagged MetH from the pJA49 plasmid (see Fig. S1F) in the *H_OFF* background. These were a wild-type MetH, a MetH^D616A^ (control of no growth [Fig. 1C; see also Fig. S4A in the supplemental material]) and a MetH^R749A^ (*metH^g2439CG>GA^*) version of the protein, which according to the results published for P. pastoris should not localize in the nucleus (34). The strain expressing wild-type MetH grew normally in restrictive conditions (see Fig. S4A), proving that the tagged MetH-GFP protein was active. Importantly, this result also demonstrated that genetic downregulation of *metH* is the only reason for the lack of growth of the *H_OFF* strain in the presence of Dox. We confirmed that A. fumigatus MetH localizes in both the nucleus and the cytoplasm (Fig. 4D; see also Fig. S4B in the supplemental material). In contrast to what was described in P. pastoris, the MetH^R749A^ protein seems to be active, since it could trigger growth of *H_OFF* in restrictive conditions (see Fig. S4A) and still localized into the nucleus (see Fig. S4B). Therefore, the possibility that MetH localization in the nucleus is important needs further exploration.

10.1128/mBio.01985-20.6 FIG S4 **(**A) Expression of MetH-GFP wild-type and MetH^R742A^-GFP strains reconstituted growth of the *H_OFF* strain in restrictive conditions, demonstrating that the proteins are active. As expected, the inactive protein MetH^D616A^-GFP did not trigger growth. (B) Western blot of MetH. Strains expressing MetH-GFP wild-type and MetH^R742A^-GFP strains were grown in the presence of Dox, nuclei were isolated from the mycelia (as described in Materials and Methods), and proteins were purified and blotted with an anti-GFP antibody. The MetH^R742A^-GFP protein could be detected in nuclei, at levels similar to those of the wild-type MetH-GFP. Download FIG S4, TIF file, 2.3 MB. Copyright © 2020 Scott et al. 2020 Scott et al. This content is distributed under the terms of the Creative Commons Attribution 4.0 International license.

### Repression of methionine synthase causes growth inhibition in growing mycelia.

**FIG 5:**
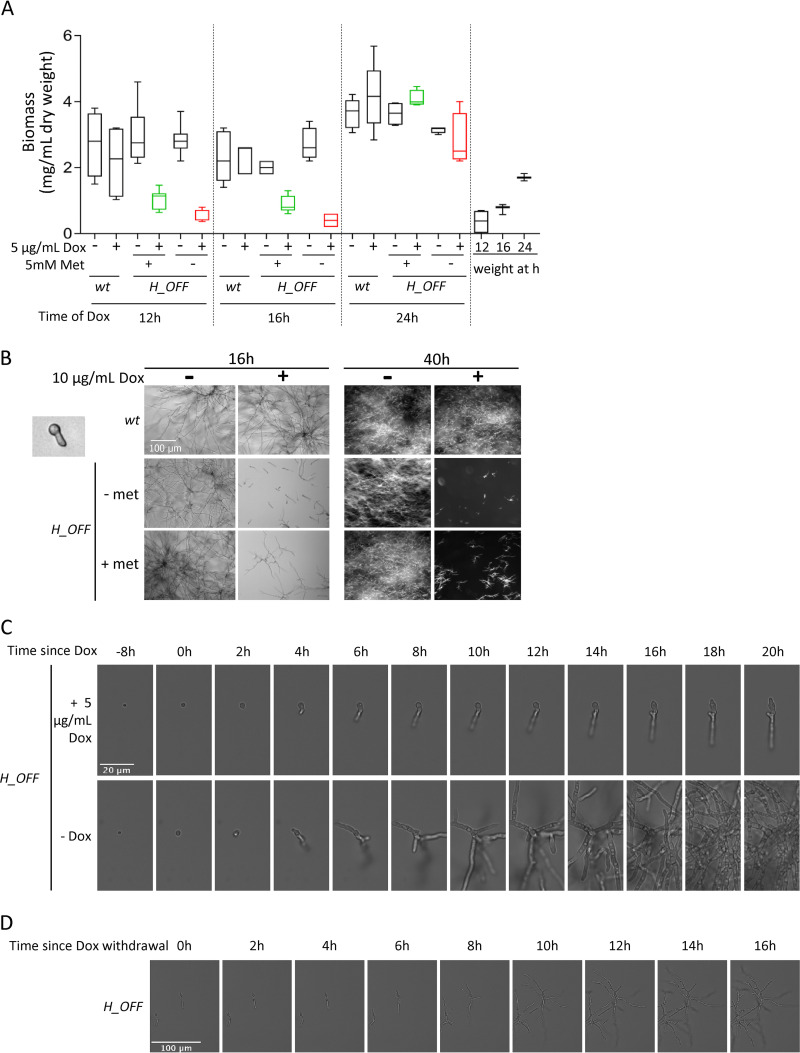
Repression of *metH* transcription causes inhibition of growth *in vitro.* (A) Addition of 1 μg/ml Dox to mycelia grown for 12 and 16 h grown strongly reduced fungal biomass, as measured 24 h later. This was more pronounced in media without methionine. The effect was lost when Dox was added 24 h after inoculation. For comparison, fungal biomass of mycelia harvested at the time of Dox addition (12, 16, and 24 h postinoculation) is shown. Three independent experiments were performed, using three technical replicates for each. (B) Microscopic images of 8-h-germinated spores treated with 10 μg/ml Dox. Images were taken 16 h after Dox addition (wide-field microscopy) and again 24 h later (stereomicroscopy). Dox addition halts growth of *H_OFF* strain in a sustained manner. (C) Time-lapse microscopy of *H_OFF* growth upon Dox addition. Dox was added to 8-h-grown conidia, which caused growth inhibition that was obvious after ∼4 h. Growth was virtually halted for as long as Dox was present. (D) At ∼6 h after Dox withdrawal, *H_OFF* growth resumed, showing that the effect was fungistatic.

The major advantage of the tetOFF system is that it can be used to simulate a drug treatment before a specific chemical is developed. The addition of Dox to a growing mycelium downregulates the gene of interest (see Fig. S1A), mimicking the effect of blocking its product by the action of a drug. The validity of the Tet systems has recently been questioned, since it has been reported that Dox can impair mitochondrial function in various eukaryotic models (70). Nevertheless, existing evidence suggests that low concentrations of Dox (≤50 μg/ml) have little effect on fungal cells. For instance, in contrast to reports of Dox affecting proliferation of human cells at low concentrations (70, 71), we and others have not detected negative effects of low concentrations of Dox or of tetracycline on fungal proliferation (Fig. 5A), phenotype (macroscopic or microscopic; Fig. 5B), or virulence (17, 72–78). In addition, it was reported that 40 μg/ml of Dox does not affect the transcriptional profile of S. cerevisiae (79), which contrasts with the broad effect caused by only 1 μg/ml Dox on global transcription in human cells (70). Moreover, a study that investigated the role of various mitochondrial proteins for its function in C. albicans did not observe any negative effect of 20 μg/ml Dox on fungal growth nor on mitochondrial morphology and function (80). To test the effect of Dox on A. fumigatus, we grew the wild-type strain overnight in various concentrations of drug and imaged mitochondria using the Rhodamine 123 dye (see Fig. S5 in the supplemental material). It was previously described that inhibition of translation in mitochondria (mechanism of Dox toxicity) promotes mitochondrial fission, which can be detected as a more fragmented, punctuate, mitochondrial appearance compared to the healthy tubular morphology (70, 80). This fragmented phenotype started to appear, although there was variation among hyphae, when the fungus was incubated in 100 μg/ml Dox and became obvious when it was incubated in 1,000 μg/ml Dox (see Fig. S5). In contrast, in low concentrations of Dox (1 and 10 μg/ml Dox) the mitochondria showed a healthy tubular morphology, indistinguishable from that of no Dox (see Fig. S5). Therefore, low concentrations of Dox do not affect mitochondria morphology and thus likely do not impair their function. In fact, we have also observed that addition of 5 μg/ml Dox to wild-type mycelium did not affect the ATP content (Fig. 3B), further supporting the conclusion that mitochondrial function is not impaired. Therefore, even if higher concentrations of Dox have a negative effect on fungal cells (74, 78), its impact at low concentrations on fungal cells seems to be minimal. Consequently, we argue that as long as the concentration of Dox used is ≤50 μg/ml, the tetOFF system can be utilized to investigate the consequences of downregulating gene expression in fungal research.

10.1128/mBio.01985-20.7 FIG S5 Doxycycline can perturb A. fumigatus hyphal mitochondrial morphology at high concentrations. A. fumigatus hyphae were stained with 10 μM Rhodamine 123 after overnight incubation in 0 μg/ml (A), 1,000 μg/ml (B), 1 μg/ml (C), 10 μg/ml (D) and 100 μg/ml (E) doxycycline. Representative single plane confocal fluorescence images are displayed in panels A to E. Notice the granulation in panel B (1,000 μg/ml) and the variable response in panel E (100 μg/ml), whereas panels C (1 μg/ml) and D (10 μg/ml) display normal mitochondrial morphology. Scale bars, 10 μm. Download FIG S5, TIF file, 2.0 MB. Copyright © 2020 Scott et al. 2020 Scott et al. This content is distributed under the terms of the Creative Commons Attribution 4.0 International license.

To investigate the effect of downregulating *metH* for mycelial growth, we added Dox to 12, 16, or 24 h grown submerged mycelia and left it incubating for an additional 24 h. Addition of Dox to 12 or 16 h grown mycelia severely impaired growth of the *H_OFF* strain but not the wild type, as observed by biomass (Fig. 5A) and optical density (OD; see Fig. S6A) measurements. This effect was lost when Dox was added to 24-h-grown mycelia due to the incapacity of Dox to reach and downregulate expression in all cells within the dense mass of an overgrown mycelium. Interestingly, Dox addition to methionine free media stopped *H_OFF* growth immediately, which can be observed by comparing fungal biomass at the time of Dox addition to the measurement 24 h after Dox addition. In contrast, the fungus inoculated in methionine containing media grew a little further after Dox addition (Fig. 5A). To understand this difference, we added Dox to either resting or 8 h germinated conidia and imaged them 16 and 40 h after drug addition (Fig. 5B; see also Fig. S6B in the supplemental material). In agreement with the previous result, we observed that Dox addition in methionine free medium inhibited growth immediately: resting conidia did not germinate and germinated conidia did not elongate the germ tube. In contrast, after the addition of Dox in methionine-containing medium, most of the resting conidia were still able to germinate and some germlings could elongate the germinated tubes to form short hyphae. This suggests that the drop in ATP levels takes ca. 3 to 4 h before having an effect on growth. Importantly, once growth was inhibited, the effect was sustained for a long period, since we could not detect further growth up to 40 h postinoculation. To corroborate these observations and further determine whether the effect of growth is fungistatic or fungicidal in the long term, we performed a time-lapse analysis of the effects of adding Dox to 8 h swollen conidia and its subsequent withdrawal after 16 h of incubation in a medium containing methionine (Fig. 5C; see also Video S1 in the supplemental material). We observed that growth was inhibited ∼4 h after Dox addition and almost completely halted after 6 h, which was sustained as long as the drug was present. Upon withdrawal of Dox, growth resumes within 6 h (Fig. 5D; see also Video S1), showing that the effect of blocking MetH is fungistatic, at least with the genetic TetOFF model of *metH* repression. As expected, Dox had no effect on wild-type growth (see Video S2).

10.1128/mBio.01985-20.8 FIG S6 **(**A) Measurement of fungal growth by optical density (OD) showed that the addition of 1 μg/ml Dox 12 or 16 h after inoculation significantly reduced fungal growth at 24 and 36 h postincubation. (B) Microscopy analysis of the effect of the addition of two concentrations of Dox to resting or 8-h-germinated conidia of the *H_OFF* strain. In the absence of methionine, growth is immediately halted in a sustained manner. In the presence of methionine, conidia can germinate, and germlings elongate the growing tube before growth stops. However, once arrested, growth is halted in a sustained manner. Microimages were taken with a wide-field microscope at 16 h postinoculation and with a stereomicroscope 40 h after inoculation (to be able to capture the huge mass of fungal growth in the control conditions). Download FIG S6, TIF file, 1.0 MB. Copyright © 2020 Scott et al. 2020 Scott et al. This content is distributed under the terms of the Creative Commons Attribution 4.0 International license.

### Targeting MetH in established infections interferes with the progression of disease.

We previously used the TetON system to investigate the relevance of MetH in A. fumigatus virulence (17). In this model, *metH* gene expression was active when mice were fed with Dox, which resulted in full virulence of the *metH_tetON* strain (demonstrating that the concentration of Dox reached in murine tissues does not impact fungal virulence). In the absence of Dox the gene was not expressed, which completely abrogated virulence. This proved that the murine lung does not readily provide the conditions to overcome the conditional essentiality of *metH*, and thus this gene is required to establish infection. However, antifungal drugs are normally administered to treat patients who already have an established infection. Therefore, it is possible that the conditional essentiality of the gene could be overcome when the fungus is actively growing in the tissue, since the fungal metabolic requirements and the environmental conditions are different (53, 54). Consequently, in order to achieve a rigorous target validation, it is crucial to assess the efficiency of new target candidates in established infections. The first conditional promoter system used for A. fumigatus *in vivo* was the (p)niiA (52). This system represses genetic expression of the gene of interest in the presence of ammonium, which is contained in murine serum. Accordingly, in this seminal study several genes essential *in vitro* were confirmed or refuted to be essential to initiate infection in a model of systemic (blood) infection. Currently, two more systems are in use to assess the relevance of fungal essential genes for pulmonary infection: TetON and (p)xylP. These systems can be used to either impede or permit fungal gene expression in murine lungs; but in both models, this control can only be exerted from the beginning of infection, since sufficient levels of the inducing molecule (doxycycline or xylose) must be present to activate gene expression in the control condition. Consequently, these models have been used to investigate the relevance of genes required to grow *in vitro* to initiate pulmonary infection (17, 76, 81). However, we believe these models are not adequate to determine the importance of the genes in established aspergillosis infections *in vivo*. This is because in this models complete elimination of the inducing metabolite (Dox or xylose), which is required to prevent expression of the gene of interest, would be gradual and take several hours after its withdrawal, which impedes a precise control of the time of intervention. Therefore, we aimed to optimize the use of the TetOFF system for this purpose because it can be used to downregulate gene expression in growing mycelia at a precise time. As a control for the model, we constructed a *cyp51A_tetOFFΔcyp51B* (*51A_OFF*) strain. We reasoned that the target of the azoles, first-line treatment drugs for *Aspergillus* diseases, should be the gold standard to compare any target against. This strain showed a similar behavior as *H_OFF in vitro*: as little as 0.05 μg/ml Dox prevented colony development on an agar plate (see Fig. S7A), and the addition of Dox to conidia or germlings blocked growth (see Fig. S7B).

10.1128/mBio.01985-20.9 FIG S7 **(**A) Colony formation of the strain *cyp51A_tetOFFΔcyp51B* (*51A_OFF*) is completely prevented with as little as 0.1 μg/ml on an agar plate. Phenotypic analysis was repeated in two independent experiments. (B) Microscopy analysis of the effect of the addition of two concentrations of Dox to resting or 8-h-germinated conidia of the 51A*_OFF* strain. In both cases growth is immediately blocked. We could observe bursting germlings with high doses of Dox (red arrow). (C) Dox regime applied to Galleria mellonella. A maximum of five doses (50 mg/kg) were applied, commencing either at the time of infection or 6 h later. (D) Dox regimen administered to mice. Treatment started 16 h after infection with a subcutaneous (s.c.) Dox injection (50 mg/kg) and change to Dox food. Treatment was maintained with s.c. injections every 12 h after the infection time point. The Dox concentration in the lungs was measured in a preliminary experiment with uninfected mice. Lungs were harvested 4 h after the beginning of treatment, 2 h after the third injection (on day 2), and 9 h after the fifth injection (on day 3, last injection). The concentrations were determined to range from 0.9 to 2.2 μg/ml, which is sufficient to downregulate gene expression according to the results *in vitro*. Download FIG S7, TIF file, 1.2 MB. Copyright © 2020 Scott et al. 2020 Scott et al. This content is distributed under the terms of the Creative Commons Attribution 4.0 International license.

**FIG 6:**
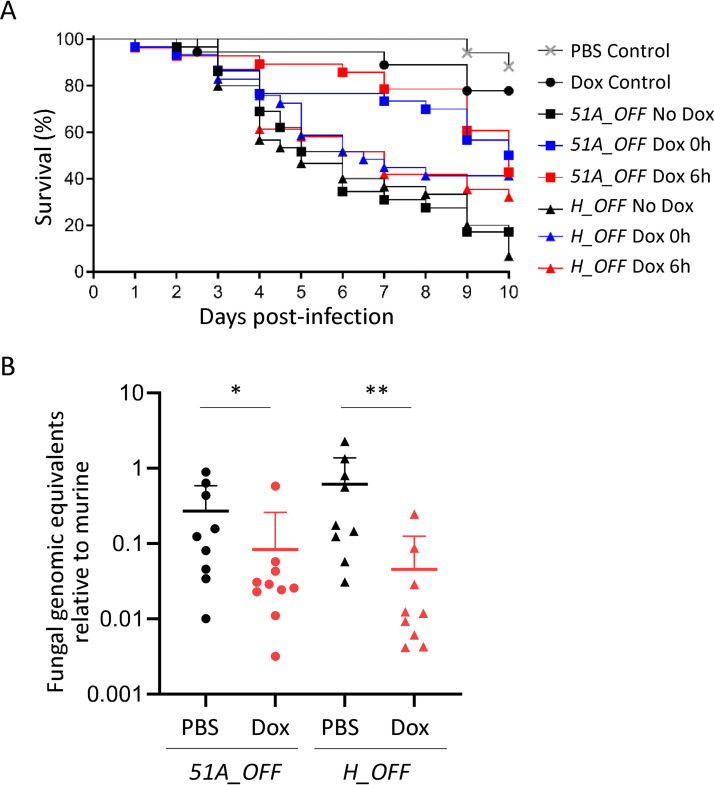
Downregulation of *metH in vivo* in established infections shows a beneficial effect comparable to the target of azoles. (A) Administration of a Dox regimen (see also Fig. S7C) to Galleria mellonella infected with either the *H_OFF* strain or the *51A_OFF* control strain showed a beneficial effect in survival. For both strains, starting regimen at the time of infection triggered a significant improvement in survival (50% versus 17.2% for *51A_OFF* [*P* = 0.0036] and 41.45% versus 6.67% for *H_OFF* [*P* = 0.022]). A Dox regimen 6 h after infection also triggered a significant improvement in survival for both strains (42.8% versus 17.2% for *51A_OFF* [*P* = 0.0007] and 32.26% versus 6.67% for *H_OFF* [*P* = 0.0324]). The curves show the pooled data from three independent experiments. Curves were compared using a log-rank test. (B) Leukopenic mice were infected with *51A_OFF* or *H_OFF* using a Dox regimen (Fig. S7D) administered 16 h after infection. Upon Dox treatment, the fungal burden in the lungs was significantly reduced for both strains (*P* = 0.0279 for *51A_OFF* and *P* = 0.0019 for *H_OFF*). Two independent experiments were carried out. Each point in the graphs represents one mouse (*n* = 9). Burdens for each strain were compared using a Mann-Whitney test.

We first assayed the use of the TetOFF system in the Galleria mellonella alternative mini-host model of infection. Preliminary experiments revealed that the balance between reaching sufficient levels of Dox to exert an effect and maintaining toxic effects of overdose low was very delicate. We finally optimized a regimen consisting of five injections of 50 mg/kg Dox (see Fig. S7C) that caused little mortality in the control group (25% in the Dox control versus 12% in the PBS treatment control *P* = 0.22) but still showed an effect of treatment (Fig. 6A). We then infected *Galleria* larvae with 5 × 10^2^ conidia of *51A_OFF* or *H_OFF* strains and applied the Dox regimen or PBS vehicle starting at the same time of infection (0 h) or 6 h after infection (see Fig. S7C). For both strains, administration of Dox from the beginning of infection triggered a significant improvement in survival compared with the nontreated conditions (50% versus 17.2% for *51A_OFF* [*P* = 0.0036] and 41.45% versus 6.67% for *H_OFF* [*P* = 0.022]) (Fig. 6A). The fact that administration of Dox at the time of infection did not improve survival to close to 100%, was not surprising, since it is important to note that Dox does not completely prevent gene expression (see Fig. S1A), so a moderate effect on survival was expectable. Furthermore, rapid metabolization of the drug in the larvae hemocoel or microenvironment variations in its concentration may also account for a discrete effect of treatment. Despite this limitation of the model, we observed that administration of Dox 6 h after infection also triggered a significant improvement in survival for both strains (42.8% versus 17.2% for *51A_OFF* [*P* = 0.0007] and 32.26% versus 6.67% for *H_OFF* [*P* = 0.0324]) (Fig. 6A). Therefore, downregulation of methionine synthase genetic expression in established infections conferred a significant benefit in survival which was comparable to that observed with the target of the azoles.

The positive results obtained using the *Galleria* infection model prompted us to assay the TetOFF system in a leukopenic murine model of pulmonary aspergillosis. To ensure that Dox levels in mouse lungs reach and maintain sufficient concentrations to downregulate gene expression (according to our results *in vitro*) we performed a pilot Dox dosage experiment in immunosuppressed noninfected mice (see Fig. S7D). We extracted the lungs of Dox-treated mice at different time points, homogenated them and measured Dox concentration using a bioassay based on inhibition of Escherichia coli BL21(DE3) growth. We could detect promising levels of Dox in all mice (concentrations ranging from 2.2 to 0.9 μg/ml; see Fig. S7D), which according to our results *in vitro* should be sufficient to downregulate gene expression from the TetOFF system. We therefore infected leukopenic mice with 10^5^ spores of the *51A_OFF* or the *H_OFF* strains and administered PBS vehicle or our Dox regimen, starting 16 h after infection (Fig. S7D). The use of an uninfected, Dox-treated control group uncovered that the intense Dox regimen used was harmful for the mice. These uninfected mice lost weight at a similar rate as the infected groups and looked ill from the third or fourth day of treatment. This is not surprising as Dox can impair mitochondrial function in mice (70) and has iron chelating properties (82). As a consequence, there was no beneficial effect of Dox treatment on survival (not shown). The fact that Dox treatment did also not show any benefit in survival for our control strain *51A_OFF*, which should mimic treatment with azoles (primary therapy for invasive aspergillosis), indicates that the TetOFF system is not ideal to mimic a drug treatment in established infections. Nevertheless, we further attempted to determine the efficiency of targeting MetH in established infections by measuring fungal burdens in lungs of treated and untreated mice. We observed that two and a half days of Dox treatment (when the mice have not developed visible toxic effects yet) did result in a significant reduction of fungal burdens 3 days after infection for both *51A_OFF* (*P* = 0.0279) and *H_OFF* (*P* = 0.0019) (Fig. 6B). Therefore, we could observe a beneficial effect of interfering with methionine synthase genetic expression in an established pulmonary infection, which was comparable to that of interfering with the expression of *cyp51A*, the target of azoles. This constitutes a very rigorous validation of MetH as a promising antifungal target.

A recent study also aimed to use another TetOFF system to validate a drug target in established aspergillosis infections (83). These authors administered Dox exclusively through oral gavage, accounting for lower dosage of drug. Consequently, even if no toxic effect for the mice was observed, they also did not detect any beneficial effect on survival when the Dox treatment was initiated after infection. Therefore, the TetOFF system is clearly not optimal and better models are needed. Yet, it is currently the only model with which the efficiency of new targets can be tested in aspergillosis established infections, and thus it is highly valuable that we have been able to optimize its use *in vivo*. Our *51A_OFF* control strain has been key to calibrating the model and allows us to be confident that the beneficial effects observed, even if subtle, are significant. Hence, we propose that using our model to gain information about the relevance of prospective molecular targets in established infections would be a valuable addition for a more comprehensive genetic validation of antifungal targets.

### Structure-based virtual screening of MetH.

**FIG 7:**
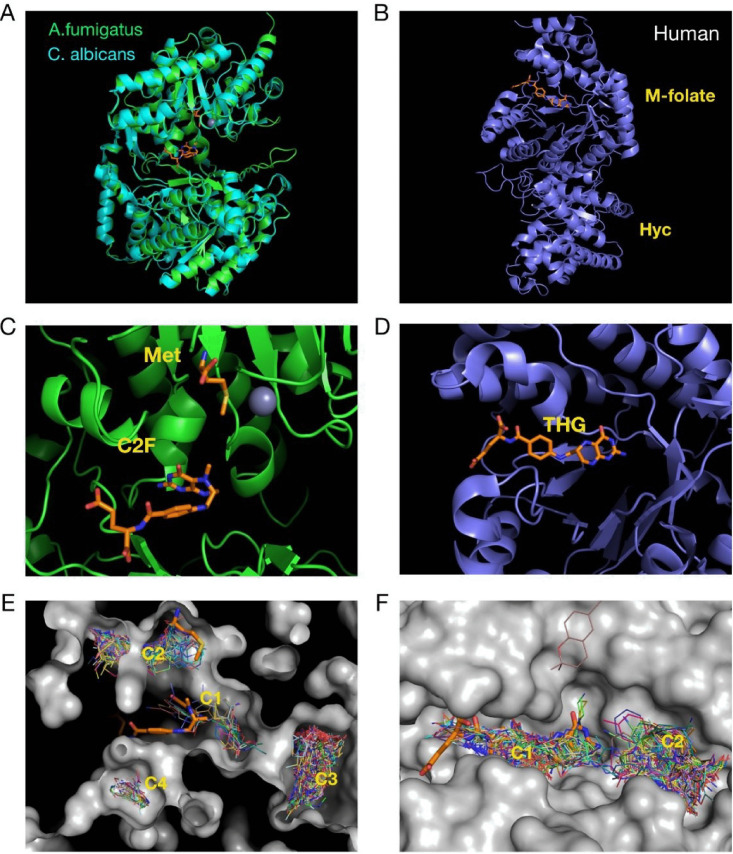
Virtual screening of fungal and human methionine synthases reveals different druggabilities of the proteins. (A) The structures of the crystallized C. albicans and the predicted A. fumigatus methionine synthases are highly similar. (B) In contrast, the structure of the human enzyme is very different, having the 5-methyl-tetrahydrafolate and homocysteine binding sites separated. (C) Detail of the active site of fungal methionine synthases, with the methionine, folate, and zinc displayed. (D) Tetrahydrofolate binding site of the human protein. (E) Virtual screening on the A. fumigatus protein found four ligand binding clusters in the structure, two of which (C1 and C2) match the binding position of the 5-methyl-tetrahydrafolate and the methionine. (F) In the human enzyme, two clusters were found, one (C1) that overlaps with the tetrahydrofolate binding site and (another C2) in a nearby pocket.

Having shown *in vivo* that MetH is a promising target, we decided to investigate its druggability by running a structure-based virtual screening. The sequence of A. fumigatus MetH (AfMetH) contains two predicted methionine synthase domains with a β-barrel fold conserved in other fungal and bacterial enzymes. The structure of the C. albicans orthologue (84) (CaMetH) showed that the active site is located between the two domains where the methyl tetrahydrofolate, the homocysteine substrate and the catalytic zinc ion bind in close proximity. The homology model for AfMetH (Fig. 7A) overlaps very well with that of the CaMetH thus providing a suitable molecular model for further analysis. In contrast, the structure of the human methionine synthase (hMS) shows a very different overall arrangement with the folate and homocysteine binding domains located in completely different regions (Fig. 7B). Comparison of the tetrahydrofolate binding sites between the fungal and the human structures also highlights significant structural differences that affect the conformation adopted by the ligand. In the CaMetH structure the 5-methyl-tetrahydrofolate (C2F) adopts a bent conformation (<20 Å long), and it is in close proximity to the methionine product, whereas in the human structure the tetrahydrofolate (THF) ligand binds in an elongated conformation extending up to 30 Å from end to end (Fig. 7C and D).

Virtual screening (VS) was carried on the AfMetH and the hMS structures with the Maybridge Ro3 fragment library to explore potential venues for drug development. The results showed four ligand binding clusters in the AfMetH structure, two of which (C1 and C2) match the binding position of the 5-methyl-tetrahydrafolate and the methionine from the CaMetH crystal structure (Fig. 7E). For the hMS, we found two main clusters, C1 that overlaps with the tetrahydrofolate binding site and C2 in a nearby pocket. Clearly, the distribution of the clusters defines a very different landscape around the folate site between the human and the fungal enzymes. Furthermore, the proximity of the C1 and C2 clusters, matching the folate and Met/homocysteine binding sites in the Ca/Af proteins means that it may be possible to combine ligands at both sites to generate double-site inhibitors with high specificity toward the fungal enzymes. Antifolates are a class of drugs that antagonize folate, blocking the action of folate dependent enzymes such as dihydrofolate reductase (DHFR), thymidylate synthase or methionine synthase. Methotrexate is an antifolate commonly used to treat cancer and autoimmune diseases. Interestingly, methotrexate has been shown to be a weak inhibitor of the C. albicans methionine synthase (33) and to have some antifungal activity against C. albicans (85) and *Aspergillus* spp. (86). Nevertheless, methotrexate is not a good antifungal drug, as its activity is high against human enzymes (50% inhibitory concentration [IC_50_] of 0.3 μM for DHFR [87]) and low against fungal methionine synthase (IC_50_ of 4 mM for C. albicans MetH [33]). Therefore, more potent and specific inhibitors of fungal methionine synthases are needed to fully exploit the value of this target for antifungal therapy, a task that seems possible and can be directed from our analyses.

In summary, we have shown that methionine synthase blockage triggers not only methionine auxotrophy but also a metabolic imbalance that results in a drop in cellular energetics and growth arrest. In light of our results, we stress that conditional essentiality is important to understand the underlying mechanisms of metabolic processes and needs to be considered to achieve proper validation of novel antimicrobial targets. Accordingly, we proved that targeting methionine synthase in established infections has a beneficial effect similar to that observed for the target of azoles, the most effective drugs for the treatment of aspergillosis. Finally, we showed that fungal methionine synthases have distinct druggable pockets that can be exploited to design specific inhibitors. In conclusion, we have demonstrated that fungal methionine synthases are promising targets for the development of novel antifungals.

## MATERIALS AND METHODS

### Strains, media, and culture conditions.

The Escherichia coli strain DH5α (88) was used for cloning procedures. Plasmid-carrying E. coli strains were routinely grown at 37°C in Luria-Bertani (LB) liquid medium (Oxoid) under selective conditions (100 μg ml^−1^ ampicillin or 50 μg ml^−1^ kanamycin); for growth on plates, 1.5% agar was added to solidify the medium. All plasmids used in the course of this study were generated using Seamless Cloning (Invitrogen) technology, as previously described (17, 89). E. coli strain BL21(DE3) (90) was grown on Mueller-Hinton agar (Sigma) in bioassays to determine Dox concentrations within homogenized murine lungs.

The wild-type clinical isolate Aspergillus fumigatus strain ATCC 46645 served as reference recipient. A. fumigatus strain A1160 (*ku80Δ*) (91) was also used to confirm *metH* essentiality. A. fumigatus mutants were generated using a standard protoplasting protocol (92). A. fumigatus strains were generally cultured in minimal medium (MM) (93) (1% glucose, 5 mM ammonium tartrate, 7 mM KCl, 11 mM KH_2_PO_4_, 0.25 mM MgSO_4_, 1× Hutner’s trace elements solution [pH 5.5], 1.5% agar) at 37°C. For selection in the presence of resistance markers 50 μg ml^−1^ of hygromycin B or 100 μg ml^−1^ of pyrithiamine (InvivoGen) were applied. In sulfur-free medium (MM–S), MgCl_2_ substituted for MgSO_4_ and a modified mixture of trace elements lacking any sulfate salt was used. For all growth assays on solid media, the culture medium was inoculated with 10 μl of a freshly prepared A. fumigatus spore suspension (10^5^ conidia ml^−1^ in water supplemented with 0.9% NaCl and 0.02% Tween 80) and incubated at 37°C for 3 days.

### Extraction and manipulation of nucleic acids.

Standard protocols of recombinant DNA technology were carried out (94). Phusion high-fidelity DNA polymerase (Thermo Fisher Scientific) was generally used in polymerase chain reactions, and essential cloning steps were verified by sequencing. Fungal genomic DNA was prepared according to the protocol of Kolar et al. (95), and Southern analyses were carried out as described previously (96, 97), using the Amersham ECL direct labeling and detection system (GE Healthcare). Fungal RNA was isolated using TRIzol reagent (Thermo Fisher Scientific) and a Qiagen plant RNA extraction kit. Retrotranscription was performed using SuperScript III first-strand synthesis (Thermo Fisher Scientific). RT-PCR on both gDNA and cDNA was performed using the SYBR Green JumpStart (Sigma) in a 7500 Fast Real Time PCR cycler from Applied Biosystems.

### Microscopy.

A total of 10^3^ A. fumigatus resting or 8 h germinated conidia were inoculated in 200 μl of medium (with or without Dox) in 8-well imaging chambers (Ibidi) and incubated at 37°C. Microscopy images were taken on a Nikon Eclipse TE2000-E, using a CFI Plan Apochromat Lambda 20×/0.75 objective, and captured with a Hamamatsu Orca-ER CCD camera (Hamamatsu Photonics) and manipulated using NIS-Elements 4.0 (Nikon). For extensively grown mycelia a stereomicroscope Leica MZFL-III was used, with a Q-imaging Retinga 6000 camera, and manipulated using Metamorph v7760. Confocal imaging was performed using a Leica TCS SP8× inverted confocal microscope equipped with a 40×/0.85 objective. Nuclei were stained with DAPI (4′,6′-diamidino-2-phenylindole; Life Technologies, Ltd.) as described previously (98). GFP was excited at 458 nm with an argon laser at 20% power. DAPI was excited at 405 nm with an LED diode at 20%.

### Metabolome analyses.

A. fumigatus wild-type and *metH_tetOFF* strains were incubated in MM for 16 h before the −Dox samples were taken (8 replicates of 11 ml each). Then, 5 μg/ml Dox and 5 mM methionine (to prevent metabolic adaptation due to met auxotrophy) were added as appropriate, and the cultures incubated for 6 h; +Dox samples were then taken (8 × 11 ml). The samples were immediately quenched with 2× volumes of 60% methanol at −48°C. After centrifugation at 4,800 × *g* for 10 min at −8°C, metabolites were extracted in 1 ml 80% methanol at −48°C by three cycles of N_2_ liquid snap-freezing, thawing, and vortexing. Supernatant was cleared by centrifugation at −9°C, 14,500 × *g* for 5 min. Quality control (QC) samples were prepared by combining 100 μl from each sample. Samples were aliquoted (300 μl), followed by the addition of 100 μl of the internal standard solution (0.2 mg/ml succinic-*d*_4_ acid and 0.2 mg/ml glycine-*d*_5_) and vortex mixing for 15 s. All samples were lyophilized by speed vacuum concentration at room temperature overnight (HETO VR MAXI vacuum centrifuge attached to a Thermo Svart RVT 4104 refrigerated vapor trap; Thermo Life Sciences, Basingstoke, UK). A two-step derivatization protocol of methoxyamination followed by trimethylsilylation was used (99).

GC-MS analysis was conducted on a 7890B GC coupled to a 5975 series MSD quadrupole mass spectrometer and equipped with a 7693 autosampler (Agilent Technologies, UK). The sample (1 μl) was injected onto a VF5-MS column (30 m × 0.25 mm × 0.25 μm; Agilent Technologies) with an inlet temperature of 280°C and a split ratio of 20:1. Helium was used as the carrier gas with a flow rate of 1 ml/min. The chromatography was programmed to begin at 70°C with a hold time of 4 min, followed by an increase to 300°C at a rate of 14°C/min and a final hold time of 4 min before returning to 70°C. The total run time for the analysis was 24.43 min. The MS was equipped with an electron impact ion source using 70 eV ionization and a fixed emission of 35 μA. The mass spectrum was collected for the range 50 to 550 *m/z* with a scan speed of 3,125 (*n *=* *1). Samples were analyzed in a randomized order with the injection of a pooled biological quality control sample after every sixth sample injection.

For data analysis, the GC-MS raw files were converted to mzXML and subsequently imported into R. The R package “erah” was used to deconvolve the GC-MS files. Chromatographic peaks and mass spectra were cross-referenced with the Golm library for putative identification purposes, and followed the metabolomics standards initiative (MSI) guidelines for metabolite identification (100). The peak intensities were normalized according to the IS (succinic-*d*_4_ acid) before being log_10_ scaled for further statistical analysis. All preprocessed data were investigated by using PCA (101).

The raw data of this metabolome analysis has been deposited in the MetaboLights database (102), under the reference MTBLS1636 (www.ebi.ac.uk/metabolights/MTBLS1636).

### ATP quantitation.

A. fumigatus was grown as in the metabolome analysis. However, where the effect of SAM was investigated spores were inoculated into MM–N + 1 mg/ml aac, and 0.5 mM SAM was also added at the time of Dox addition. ATP levels were determined using the BacTiter-Glo assay (Promega) according to the manufacturer’s instructions and a TriStar LB 941 microplate reader (Berthold).

### Isolation and detection of SAM.

A. fumigatus was grown exactly under the same conditions as described for the metabolome analysis. Harvested mycelia were snap-frozen in liquid N_2_ and stored at −70°C before SAM isolation. SAM extraction was carried out as described by Owens et al. (103). Briefly, frozen mycelia were ground in liquid N_2_, and 0.1 M HCl (250 μl) was added to ground mycelia (100 mg). Samples were stored on ice for 1 h, with sample vortexing at regular intervals. Samples were centrifuged at 13,000 × *g* for 10 min (4°C) to remove cell debris, and supernatants were collected. The concentration of protein in supernatants was determined using a Bio-Rad Bradford protein assay relative to a bovine serum albumin (BSA) standard curve. Clarified supernatants were adjusted to 15% (wt/vol) trichloroacetic acid to remove protein. After 20 min of incubation on ice, centrifugation was repeated and clarified supernatants were diluted with 0.1% (vol/vol) formic acid. Samples were injected onto a Hypersil Gold aQ C_18_ column with polar endcapping on a Dionex UltiMate 3000 nanoRSLC with a Thermo Q-Exactive mass spectrometer. Samples were loaded in 100% solvent A (0.1% [vol/vol] formic acid in water), followed by a gradient to 20% solvent B (0.1% [vol/vol] formic acid in acetonitrile) over 4 min. Resolution set to 70,000 for MS, with MS/MS scans collected using a Top3 method. SAM standard (Sigma) was used to determine retention time and to confirm MS/MS fragmentation pattern for identification. Extracted ion chromatograms were generated at *m/z* 399 to 400, and the peak area of SAM was measured. Measurements were taken from three biological and two technical replicates per sample, normalized to the protein concentration in the extracts from each replicate. SAM levels are expressed as a percentage relative to the parental strain in the absence of Dox.

### Nucleus isolation and Western blotting.

Protoplasts were generated as in A. fumigatus transformations, and nuclei were isolated by sucrose gradient fractionation as previously described by Sperling and Grunstein (104). Nuclear localization of GFP-tagged target proteins was confirmed by Western blotting. Aliquots of nuclei were boiled for 5 min in loading buffer (0.2 M Tris-HCl, 0.4 M dithiothreitol, 8% SDS, trace bromophenol blue) and separated on a 12% (wt/vol) SDS-PAGE gel. The proteins were transferred to a polyvinylidene difluoride membrane using the Trans-Blot Turbo transfer system (Bio-Rad). Detection of GFP was carried out with a rabbit polyclonal anti-GFP antiserum (Bio-Rad) and anti-rabbit IgG HRP-linked antibody (Cell Signaling Technology). SuperSignal West Pico PLUS Chemiluminescent Substrate (Thermo Scientific) and the ChemiDoc XRS+ Imaging System (Bio-Rad) were used to visualize immunoreactive bands. Ponceau S staining was performed to normalize the Western blotting signal to the protein loading.

### Mitochondrion imaging.

Approximately 200 A. fumigatus spores were seeded onto Ibidi 8-well slides in minimal medium containing 0, 1, 10, 100, and 1,000 μg/ml Dox and incubated at 37°C for 16 h. The culture medium was then replaced with minimal medium containing the same Dox concentrations plus 10 μM rhodamine-123 dye and further incubated for 1 to 2 h at 37°C prior to live-cell confocal imaging. High-resolution confocal fluorescence imaging was performed at 37°C on a Leica SP8x using a 63×/1.3 NA oil immersion lens, whereby the rhodamine-123 fluorescence was excited with a white light laser (5%) tuned to 508 nm, and the emission was collected on HyD detectors set to 513 to 600 nm. Representative single-plane images of each condition were background subtracted (rolling ball 20 pixel radius) in Fiji (105) and montaged using FigureJ (106).

### Biomass measurement.

Conidia were inoculated into MM–S, supplemented with either methionine or sulfate, and incubated at 37°C and 180 rpm for 12, 16, or 24 h. After this initial incubation, 3-ml samples were taken in triplicate from the cultures, filtered through tared Miracloth, and dried at 60°C for 16 h, and their biomass was measured. In treated conditions Dox was added to a final concentration of 1 μg/ml, and the culture was allowed to grow for a further 24 h at 37°C and 180 rpm. Then, 5-ml samples were taken in triplicate, and their biomass was measured as above.

### *Galleria mellonella* infections.

Sixth-stage instar larval G. mellonella moths (15 to 25 mm in length) were ordered from the Live Foods Company (Sheffield, United Kingdom). Infections were performed according to Kavanagh and Fallon (107). Randomly selected groups of 15 larvae were injected in the last left proleg with 10 μl of a suspension of 5 × 10^4^ conidia/ml in PBS, using Braun Omnican 50-U 100 0.5-ml insulin syringes with integrated needles. Dox was administered according to the treatment shown in Fig. S7C, alternating injections in the last right and left prolegs. In each experiment, an untouched and a saline injected control were included to verify that mortality was not due to the health status of the larvae or the injection method. Three independent experiments were carried out. The presented survival curves display the pooled data, which was analyzed with a log-rank test.

### Ethics statement.

All mouse experiments were performed under United Kingdom Home Office project license PDF8402B7 and approved by the University of Manchester Ethics Committee and by the Biological Services Facility at the Faculty of Biology, Medicine and Health, University of Manchester.

### Leukopenic murine model of invasive pulmonary aspergillosis and calculation of fungal burden.

Outbred CD1 male mice (22 to 26 g) were purchased from Charles Rivers and left to rest for at least 1 week before the experiment. Mice were allowed access *ad libitum* to water and food throughout the experiment. Mice were immunosuppressed with 150 mg/kg of cyclophosphamide on days −3 and −1 and with 250 mg/kg cortisone acetate on day −1. On day 0 mice were anesthetized with isoflurane and intranasally infected with a dose of 10^5^ conidia (40 μl of a freshly harvested spore solution of 2.5 × 10^6^ conidia/ml). Dox was administered according to the treatment shown in Fig. S7D. Dox containing food was purchased from Envingo (Safe-Diet U8200 version 0115 A03 0.625 g/kg Doxycycline Hyclate pellets). At the selected time point (72 h after infection for fungal burden), mice were sacrificed by a lethal injection of pentobarbital, and the lungs were harvested and immediately snap frozen.

Frozen lungs were lyophilized for 48 h in a CoolSafe ScanVac freeze drier connected to a VacuuBrand pump and subsequently ground in the presence of liquid nitrogen. DNA was isolated from the powder using the DNeasy blood and tissue kit (Qiagen). DNA concentration and quality were measured using a NanoDrop 2000 (Thermo Fisher Scientific). To detect the fungal burden, 500-ng portions of DNA extracted from each infected lung were subjected to qPCR. Forward (5′-ACTTCCGCAATGGACGTTAC-3′) and reverse (5′-GGATGTTGTTGGGAATCCAC-3’) primers were used to amplify the A. fumigatus β-tubulin gene (AFUA_7G00250). The primers designed to amplify the murine actin locus (NM_007393) were as follows: forward (5′-CGAGCACAGCTTCTTTGCAG-3′) and reverse (5′-CCCATGGTGTCCGTTCTGA-3′). Standard curves were calculated using different concentrations of fungal and murine gDNA pure template. Negative controls containing no template DNA were subjected to the same procedure to exclude or detect any possible contamination. Three technical replicates were prepared for each lung sample. qPCRs were performed using the 7500 Fast Real-Time PCR system (Thermo Fisher Scientific) with the following thermal cycling parameters: 94°C for 2 min and 40 cycles of 94°C for 15 s and 59°C for 1 min. The fungal burden was calculated by normalizing the number of fungal genome equivalents (i.e., the number of copies of the tubulin gene) to the murine genome equivalents in the sample (i.e., the number of copies of the actin gene) (108). Two independent experiments were carried out (*n* = 9, five mice in the first experiment and four mice in the second experiment). The burdens for each strain were compared by using a Mann-Whitney test.

### Molecular homology models and virtual screening.

The full-length sequence for AFUA_4G07360, the cobalamin-independent methionine synthase MetH from A. fumigatus (AfMetH) was obtained from FungiDB (https://fungidb.org/fungidb) (109). This sequence, together with the structure of the C. albicans orthologue (CaMetH) (PDB ID 4L65; doi:10.1016/j.jmb.2014.02.006), was used to create the molecular homology model in Modeller (v9.23) (110) with the basic option mode. The AfMetH model was then used for virtual screening with the semiautomated pipeline VSpipe (111). For comparison, we also performed virtual screening with the structure of the human methionine synthase containing the folate and homocysteine binding domains (PDB ID 4CCZ). Docking was done using the Maybridge Ro3 1000 fragment library with AutoDock Vina (112). The results were inspected graphically by using PyMOL (v1.8.0.3 Enhanced for Mac OS X; Schrödinger). All images were produced by using PyMOL.

### Data availability.

The raw data that support the findings of this study are available upon reasonable request to the authors. The raw data from the metabolome analysis have been deposited in the MetaboLights database (102) under the reference MTBLS1636 (www.ebi.ac.uk/metabolights/MTBLS1636).

10.1128/mBio.01985-20.1 VIDEO S1 Time-lapse of the growth of the *H_OFF* strain upon Dox addition and withdrawal. Download Movie S1, AVI file, 9.9 MB. Copyright © 2020 Scott et al. 2020 Scott et al. This content is distributed under the terms of the Creative Commons Attribution 4.0 International license.

10.1128/mBio.01985-20.2 VIDEO S2 Time-lapse of the growth of the wild-type strain upon Dox addition. Download Movie S2, AVI file, 13.2 MB. Copyright © 2020 Scott et al. 2020 Scott et al. This content is distributed under the terms of the Creative Commons Attribution 4.0 International license.
