# Supplementary figures and images for: Targeting Methionine Synthase in a Fungal Pathogen Causes a Metabolic Imbalance That Impacts Cell Energetics, Growth, and Virulence
Source: mBio. 2020 Oct 13;11(5):e01985-20. doi: 10.1128/mBio.01985-20 (PMC7554668; doi:10.1128/mBio.01985-20)

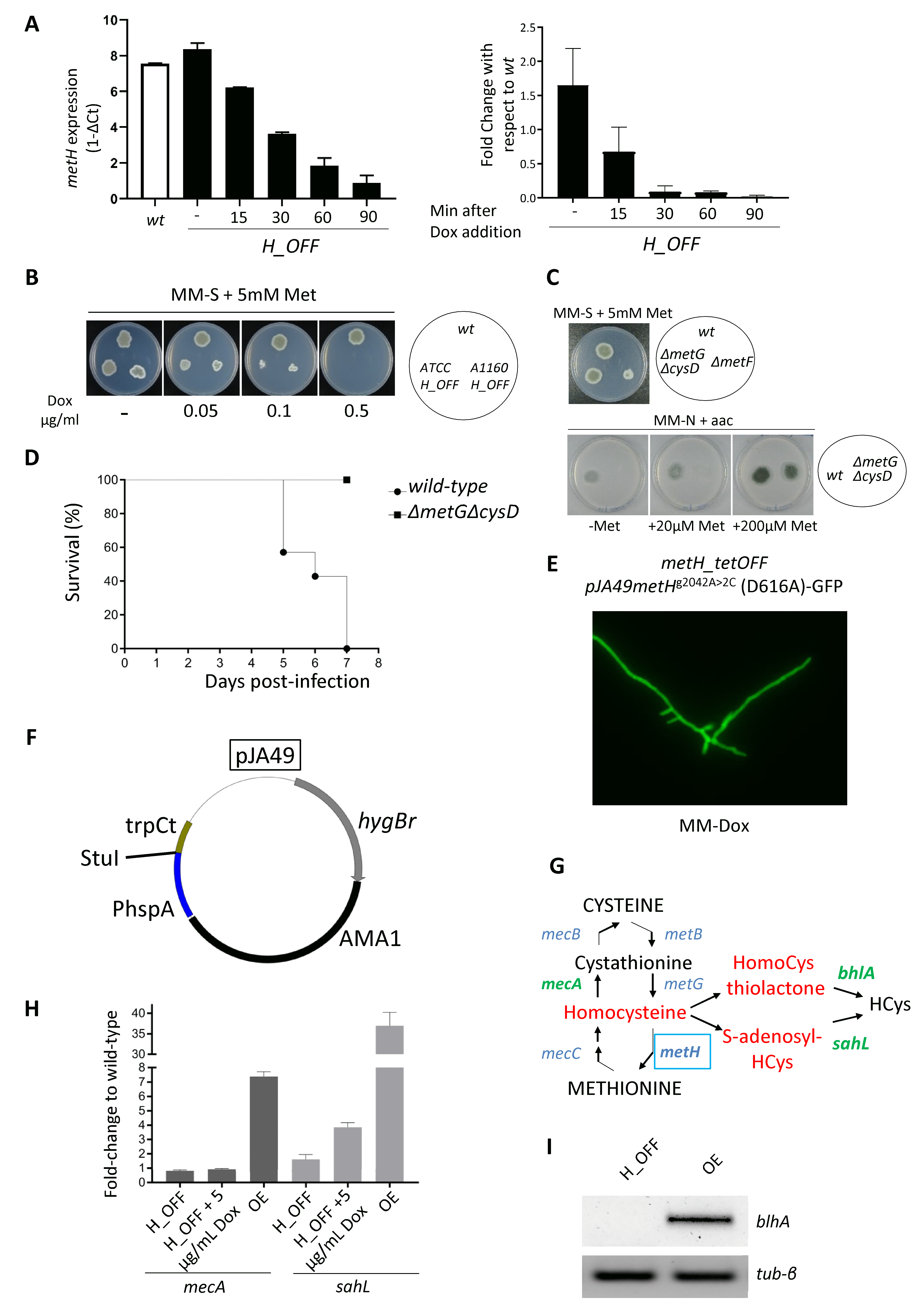

Supplement: FIG S1 [file mBio.01985-20-sf001.tif]

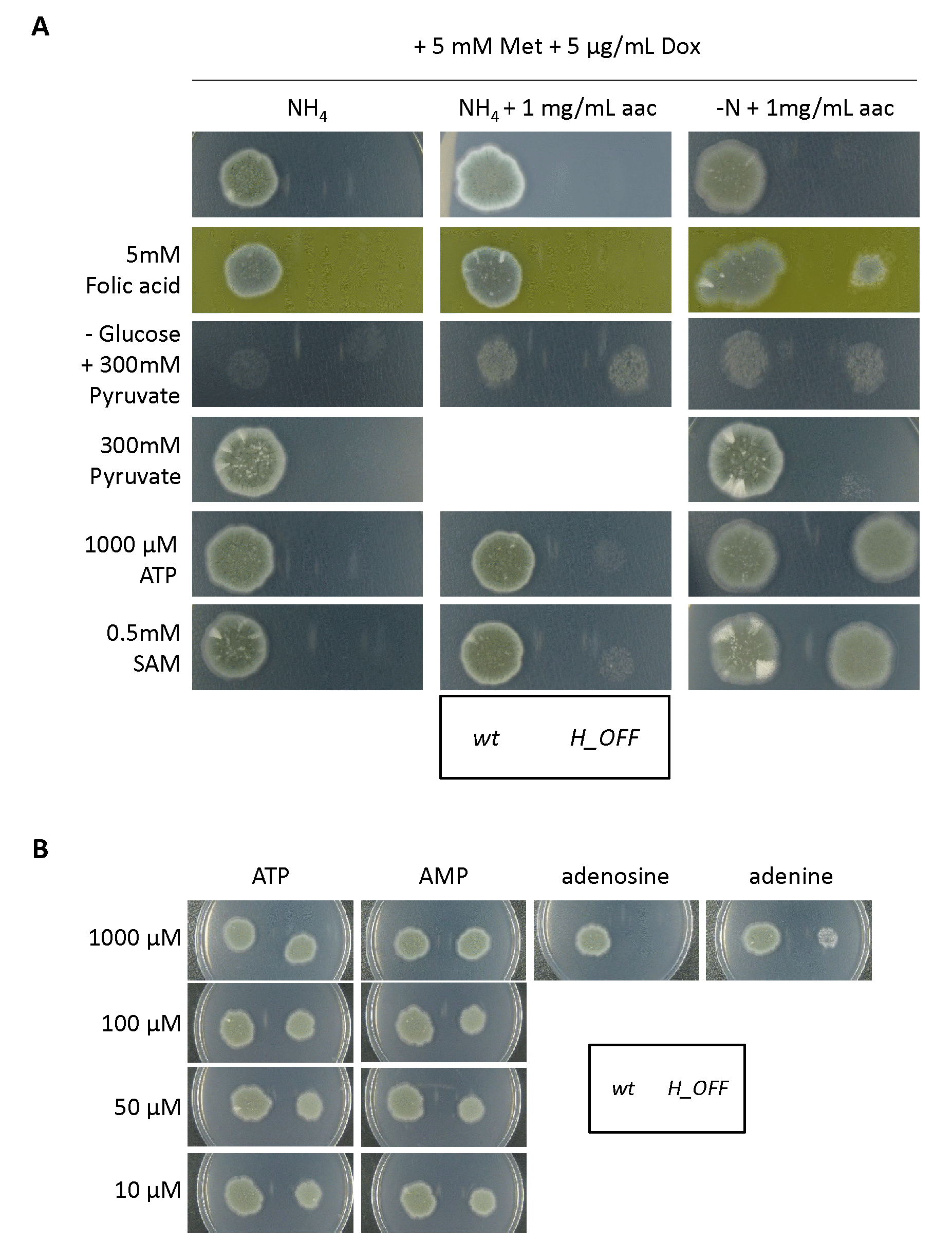

Supplement: FIG S2 [file mBio.01985-20-sf002.tif]

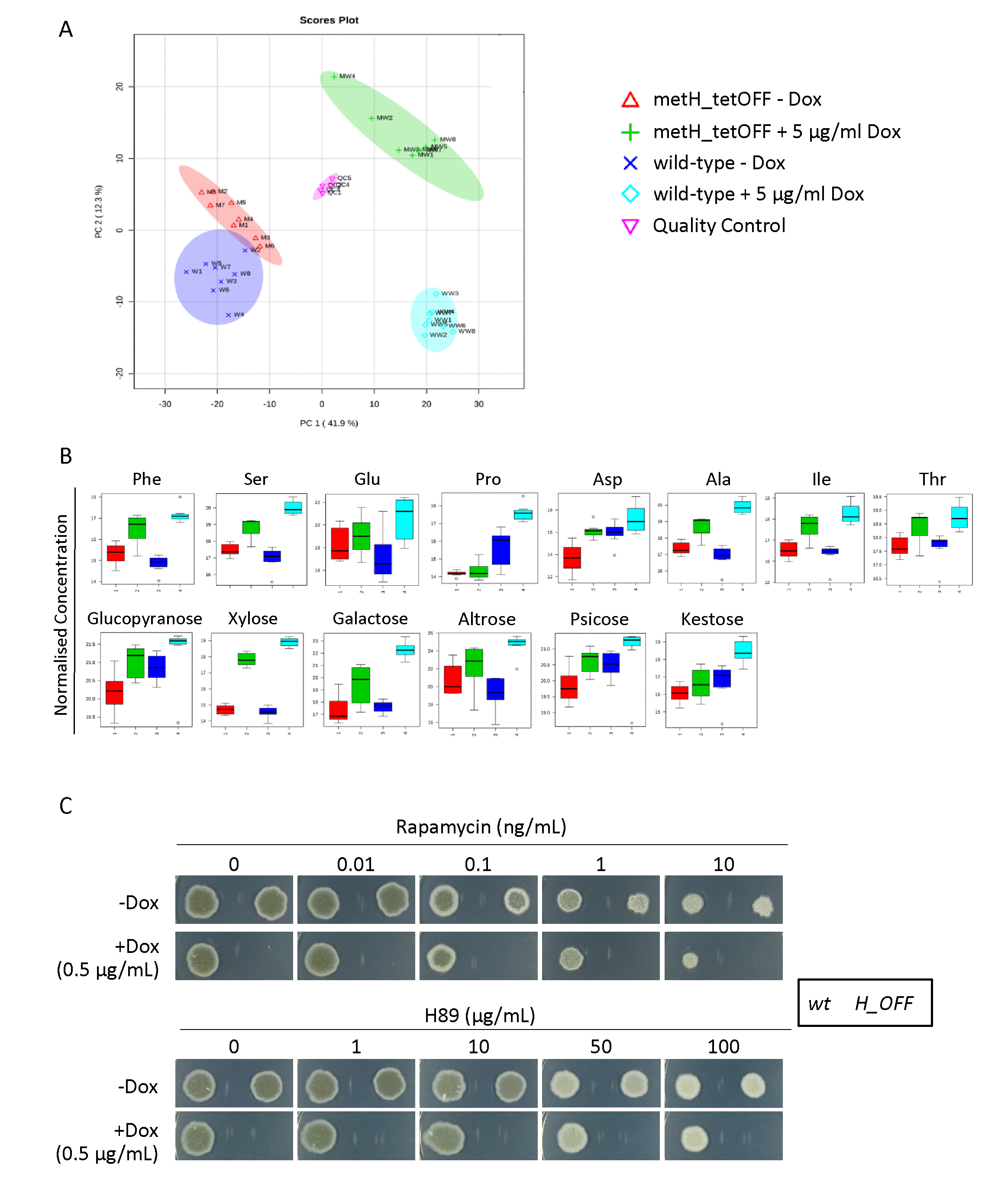

Supplement: FIG S3 [file mBio.01985-20-sf003.tif]

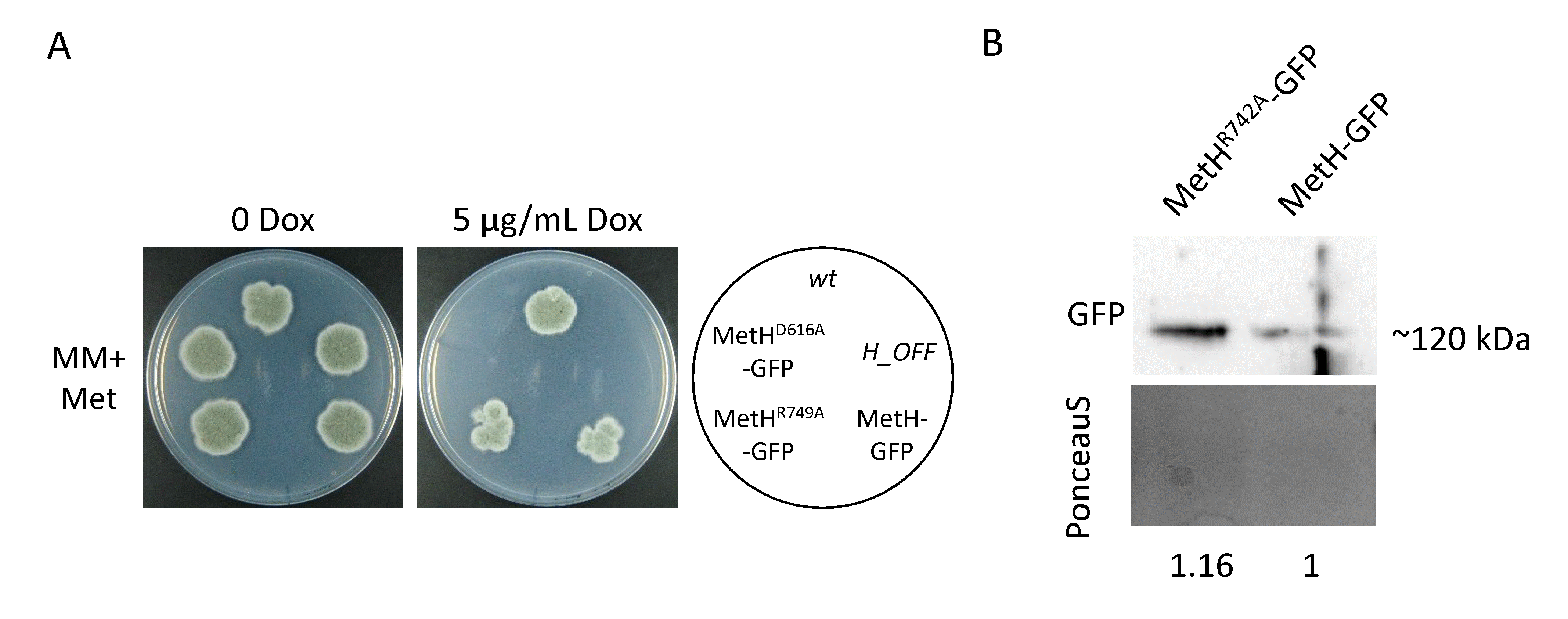

Supplement: FIG S4 [file mBio.01985-20-sf004.tif]

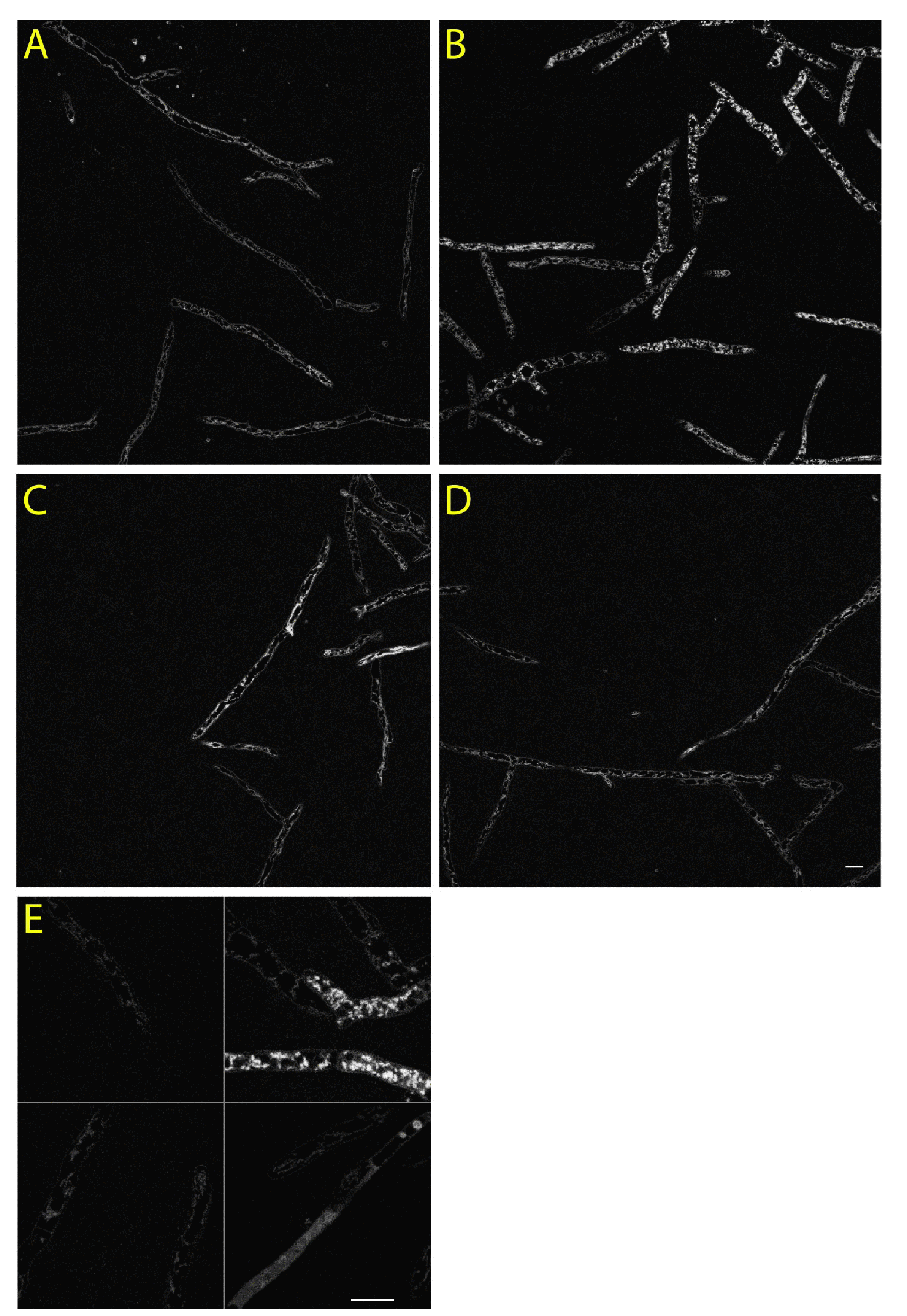

Supplement: FIG S5 [file mBio.01985-20-sf005.tif]

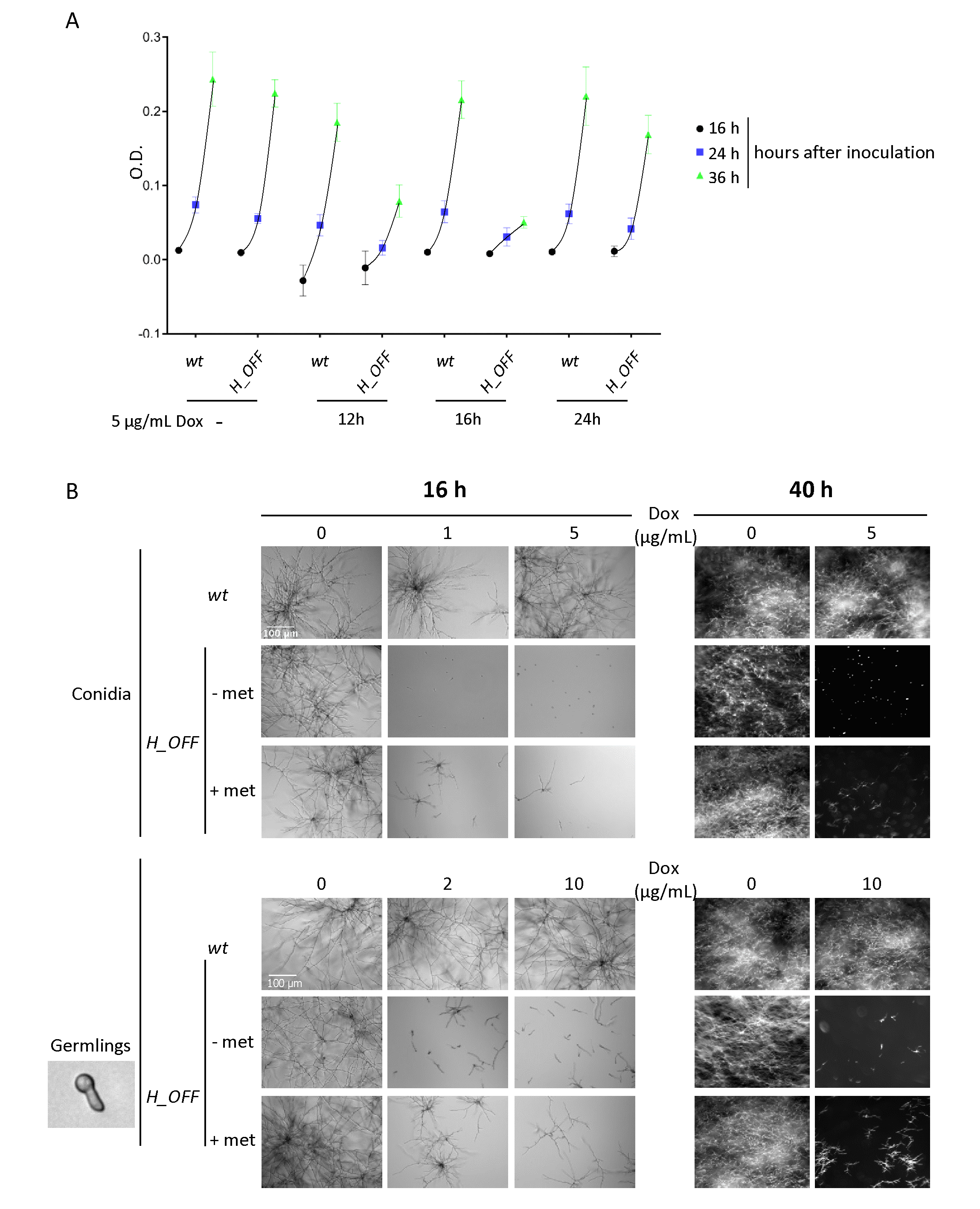

Supplement: FIG S6 [file mBio.01985-20-sf006.tif]

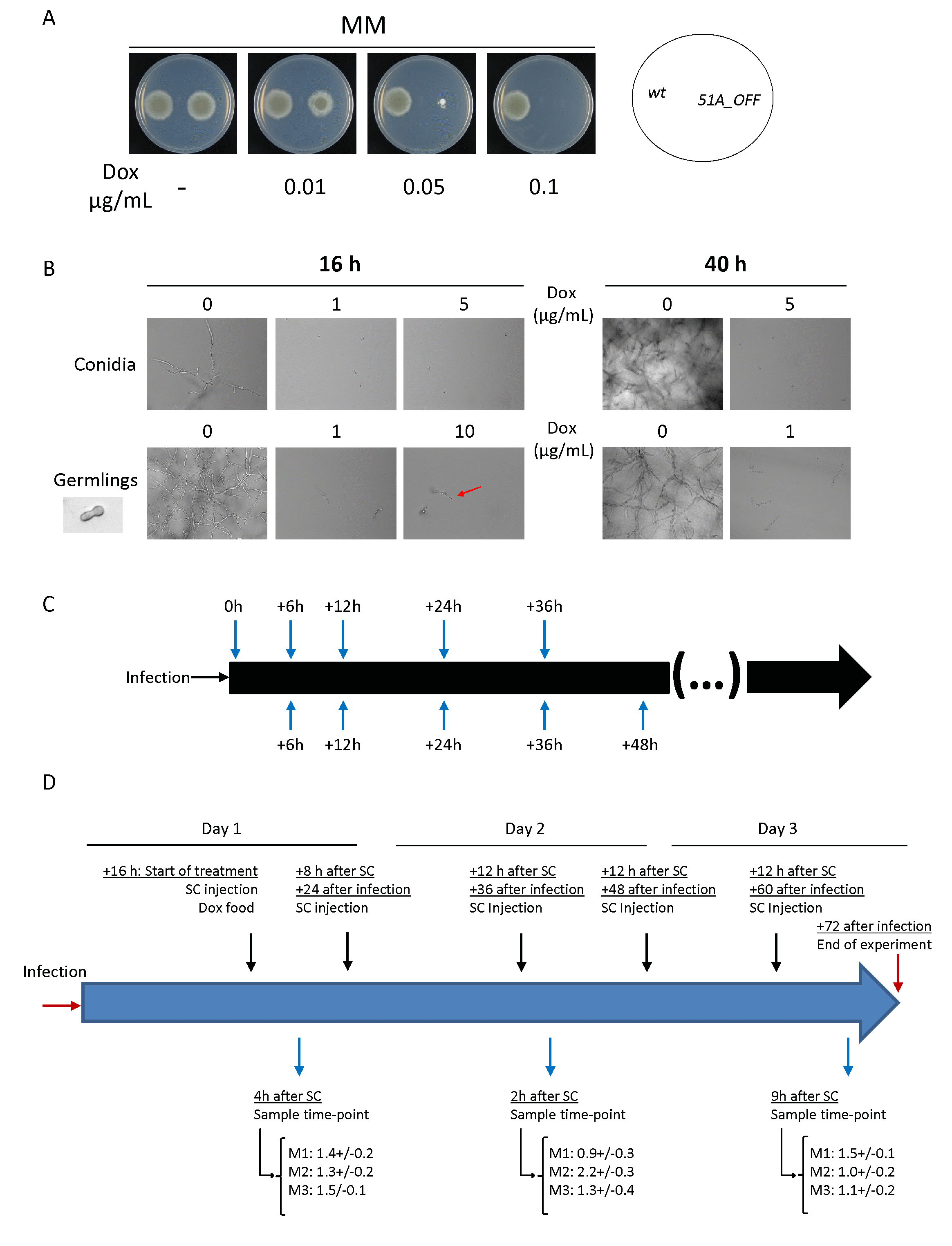

Supplement: FIG S7 [file mBio.01985-20-sf007.tif]
