# Supplementary material for: Targeting Methionine Synthase in a Fungal Pathogen Causes a Metabolic Imbalance That Impacts Cell Energetics, Growth, and Virulence
Source: mBio. 2020 Oct 13;11(5):e01985-20. doi: 10.1128/mBio.01985-20 (PMC7554668; doi:10.1128/mBio.01985-20)
Supplement: TABLE S1 [file mBio.01985-20-st001.pdf]

Number metabolite	f. value	p. value	#	FDR	ID
1	21,388	1,15E-08	7,9394	2,62E-08	NA
2	5,594	0,00157	2,8042	0,0021085	Acetic acid, aminooxy- (3TMS)
3	44,72	1,15E-12	11,939	4,23E-12	NA
4	54,449	7,87E-14	13,104	3,37E-13	NA
5	7,9211	0,000148	3,8298	0,0002186	NA
6	11,68	5,84E-06	5,234	1,01E-05	NA
7	26	1,17E-09	8,9325	2,95E-09	Homoserine (2TMS)
8	14,425	7,77E-07	6,1096	1,48E-06	NA
9	87,913	8,60E-17	16,066	5,77E-16	Hydrogen sulfide (2TMS)
10	14,816	5,95E-07	6,2258	1,14E-06	Carbodiimide (2TMS)
11	31,282	1,20E-10	9,9197	3,57E-10	NA
13	35,971	2,03E-11	10,692	6,29E-11	NA
15	23,185	4,54E-09	8,3432	1,08E-08	Glyoxylic acid (1MEOX) (1TMS)
18	5,1069	0,002684	2,5712	0,0035393	Dodecane
19	37,451	1,20E-11	10,92	3,90E-11	Proline (1TMS)
20	99,317	1,44E-17	16,842	1,09E-16	Alanine, beta- (1TMS)
21	14,834	5,87E-07	6,2311	1,14E-06	Pyridine, 2-hydroxy- (1TMS)
22	36,043	1,98E-11	10,704	6,18E-11	Pyruvic acid (1MEOX) (1TMS)
23	11,341	7,62E-06	5,118	1,29E-05	Lactic acid (2TMS)
24	4,237	0,007281	2,1378	0,0092577	Dodecane
25	7,9058	0,00015	3,8235	0,0002209	Dodecane
26	4,1472	0,008095	2,0918	0,010256	Dodecane
28	18,375	6,23E-08	7,2052	1,35E-07	Pyruvic acid (2TMS)
29	22,565	6,21E-09	8,2066	1,44E-08	Valine (1TMS)
30	26,041	1,15E-09	8,9406	2,92E-09	Putrescine, N-methyl- (3TMS)
31	55,472	6,08E-14	13,216	2,74E-13	Sarcosine (2TMS)
33	4,9922	0,003053	2,5153	0,0040102	Dodecane
34	6,0651	0,000948	3,0232	0,0012981	Dodecane
36	63,369	9,45E-15	14,025	4,80E-14	Isovaleric acid, 2-oxo- (1MEOX) (1TMS
37	23,316	4,25E-09	8,3719	1,02E-08	Hydroxylamine (3TMS)
38	22,735	5,70E-09	8,2442	1,33E-08	NA
42	3,3896	0,020222	1,6942	0,02457	NA
44	8,2996	0,000104	3,9841	0,0001565	NA
46	3,4306	0,019228	1,7161	0,023442	NA
47	17,918	8,19E-08	7,0868	1,76E-07	NA
48	8,7857	6,64E-05	4,1776	0,0001019	Carbonic acid (1MEOX) (2TMS)
49	7,9988	0,000137	3,8617	0,0002044	Carbonic acid (1MEOX) (2TMS)
50	542,88	6,56E-29	28,183	1,17E-26	NA
51	349,84	6,48E-26	25,188	3,84E-24	NA
52	67,53	3,84E-15	14,416	2,07E-14	NA
54	50,212	2,40E-13	12,621	9,69E-13	Ethanolamine (3TMS)
55	14,331	8,29E-07	6,0813	1,57E-06	NA
56	13,952	1,08E-06	5,9661	2,02E-06	Unknown#bth-pae-013
57	3,1285	0,027967	1,5534	0,033636	NA
58	11,004	9,98E-06	5,0008	1,68E-05	NA
59	281,46	1,92E-24	23,717	8,54E-23	NA

61	9,6911	2,99E-05	4,525	4,70E-05	NA
62	131,95	2,09E-19	18,68	2,19E-18	NA
63	57,077	4,09E-14	13,388	1,92E-13	NA
64	22,736	5,69E-09	8,2445	1,33E-08	Isoleucine (1TMS)
65	5,4279	0,001881	2,7255	0,0025086	NA
68	8,7796	6,68E-05	4,1752	0,0001021	NA
70	41,622	3,00E-12	11,522	1,02E-11	NA
73	7,0974	0,000329	3,4825	0,000467	NA
76	4,982	0,003088	2,5103	0,0040417	Valine (2TMS)
77	25,986	1,18E-09	8,9296	2,95E-09	NA
78	69,704	2,45E-15	14,612	1,38E-14	NA
79	238,89	2,43E-23	22,614	6,67E-22	NA
80	167,48	5,67E-21	20,247	7,76E-20	NA
81	22,653	5,94E-09	8,2261	1,38E-08	Butanoic acid, 4-hydroxy- (2TMS)
82	69,401	2,60E-15	14,584	1,45E-14	NA
83	56,543	4,66E-14	13,332	2,15E-13	Unknown#sst-cgl-008
84	40,069	4,97E-12	11,304	1,65E-11	1,3-Dihydroxyaceton (1MEOX) (2TMS
86	46,562	6,68E-13	12,175	2,59E-12	Serine (2TMS)
87	46,303	7,21E-13	12,142	2,76E-12	Serine (2TMS)
88	44,556	1,21E-12	11,917	4,40E-12	Alanine (2TMS)
92	4,2739	0,006973	2,1566	0,0088969	Alkane
93	18,825	4,79E-08	7,3199	1,05E-07	Phosphoric acid (3TMS)
94	2,8483	0,039788	1,4002	0,047373	Phosphoric acid (3TMS)
95	20,155	2,25E-08	7,6481	5,07E-08	Phosphoric acid (3TMS)
96	4,5169	0,005252	2,2797	0,0067743	NA
101	9,4132	3,80E-05	4,4201	5,96E-05	Threonine (2TMS)
103	18,172	7,03E-08	7,1529	1,52E-07	similar to Cyclohexasiloxane, dodecan
105	8,2778	0,000106	3,9753	0,000159	NA
107	6,5015	0,000601	3,2208	0,0008332	NA
110	30,865	1,42E-10	9,8466	4,12E-10	Succinic acid (2TMS)
112	5,3933	0,001954	2,709	0,0025961	NA
113	258,45	7,20E-24	23,142	2,80E-22	Glyceric acid (3TMS)
114	220,55	8,35E-23	22,078	1,75E-21	NA
115	7,4496	0,000233	3,633	0,0003342	Alkane
119	58,534	2,88E-14	13,541	1,37E-13	NA
120	53,473	1,01E-13	12,996	4,23E-13	NA
121	37,837	1,05E-11	10,978	3,44E-11	NA
122	16,616	1,83E-07	6,7382	3,78E-07	Alanine (3TMS)
123	172,12	3,74E-21	20,428	5,54E-20	NA
124	229,05	4,66E-23	22,332	1,19E-21	Mevalonic acid-1,5-lactone (1TMS)
125	56,028	5,29E-14	13,276	2,42E-13	NA
126	74,328	9,76E-16	15,011	5,70E-15	NA
127	4,0579	0,009	2,0458	0,011321	Tryptamine, 5-hydroxy- (4TMS)
128	12,572	2,95E-06	5,5306	5,25E-06	Alanine [+CO2] (2TMS)
130	43,317	1,77E-12	11,753	6,16E-12	NA
131	14,086	9,84E-07	6,0071	1,84E-06	Aspartic acid (2TMS)
133	120,62	8,03E-19	18,095	7,33E-18	Alanine, beta- (3TMS)

134	96,471	2,21E-17	16,656	1,60E-16	NA
135	87,989	8,49E-17	16,071	5,77E-16	NA
136	158,26	1,34E-20	19,873	1,70E-19	Tyrosine, 3-iodo- (3TMS)
138	13,157	1,91E-06	5,7184	3,47E-06	NA
140	113,08	2,10E-18	17,677	1,83E-17	NA
141	30,784	1,47E-10	9,8323	4,22E-10	NA
142	54,683	7,41E-14	13,13	3,26E-13	NA
143	121,72	7,01E-19	18,154	6,57E-18	NA
144	43,996	1,43E-12	11,844	5,16E-12	Malic acid (3TMS)
147	180,1	1,87E-21	20,728	3,03E-20	Threitol (4TMS)
148	180,6	1,79E-21	20,746	3,03E-20	NA
149	16,456	2,02E-07	6,6941	4,14E-07	Alkane
150	6,8157	0,000437	3,3599	0,000612	NA
154	16,425	2,06E-07	6,6856	4,20E-07	Pyroglutamic acid (1TMS)
155	24,758	2,10E-09	8,6787	5,15E-09	Pyroglutamic acid (1TMS)
157	4,0902	0,008661	2,0624	0,010934	Butanoic acid, 4-amino- (3TMS)
158	12,822	2,45E-06	5,6115	4,42E-06	NA
159	3,6839	0,014107	1,8506	0,017498	Glutamic acid (2TMS)
160	3,0674	0,030191	1,5201	0,036068	Glutamic acid (2TMS)
161	6,0481	0,000965	3,0154	0,0013164	NA
163	9,7763	2,77E-05	4,5568	4,39E-05	NA
166	10,827	1,15E-05	4,9383	1,91E-05	NA
168	176,78	2,48E-21	20,605	3,84E-20	NA
169	22,362	6,90E-09	8,1612	1,58E-08	NA
172	32,575	7,22E-11	10,141	2,18E-10	Alkane
173	31,11	1,29E-10	9,8896	3,76E-10	Phenylalanine (1TMS)
174	49,943	2,58E-13	12,589	1,03E-12	NA
175	601,32	1,31E-29	28,883	4,66E-27	NA
176	43,833	1,51E-12	11,822	5,36E-12	Glutaric acid, 2-hydroxy- (3TMS)
177	221,9	7,60E-23	22,119	1,69E-21	NA
181	134,58	1,55E-19	18,808	1,74E-18	NA
182	219,22	9,16E-23	22,038	1,81E-21	Glutaric acid, 2-oxo- (1MEOX) (2TMS)
183	11,782	5,39E-06	5,2686	9,40E-06	NA
185	51,877	1,53E-13	12,815	6,27E-13	NA
187	65,114	6,44E-15	14,191	3,37E-14	NA
188	68,029	3,46E-15	14,461	1,89E-14	NA
189	12,591	2,91E-06	5,5368	5,20E-06	NA
191	113,6	1,97E-18	17,707	1,75E-17	NA
194	42,91	2,00E-12	11,698	6,92E-12	Tartaric acid (4TMS)
195	38,447	8,55E-12	11,068	2,82E-11	NA
196	7,8286	0,000162	3,7916	0,0002348	NA
197	7,6713	0,000188	3,7262	0,0002708	NA
199	7,7526	0,000174	3,76	0,0002515	Benzoic acid, 4-hydroxy- (2TMS)
200	6,9077	0,000398	3,4002	0,0005622	Unknown#sst-cgl-044
201	7,8476	0,000159	3,7995	0,0002315	Gluconic acid-1,4-lactone (4TMS)
202	58,606	2,83E-14	13,548	1,36E-13	NA
204	309,16	4,46E-25	24,351	2,27E-23	Xylose (1MEOX) (4TMS) BP

205	27,268	6,57E-10	9,1825	1,72E-09	Arabitol (5TMS)
206	89,064	7,11E-17	16,148	4,96E-16	NA
208	109,38	3,45E-18	17,463	2,92E-17	Arabitol (5TMS)
209	17,288	1,20E-07	6,9203	2,53E-07	Galactitol (6TMS)
211	6,0649	0,000948	3,0232	0,0012981	Alkane
212	145,75	4,66E-20	19,331	5,54E-19	NA
214	19,653	2,98E-08	7,5261	6,58E-08	NA
215	74,576	9,30E-16	15,031	5,52E-15	NA
216	65,402	6,05E-15	14,218	3,21E-14	NA
217	9,0808	5,10E-05	4,2927	7,89E-05	NA
218	134,54	1,56E-19	18,807	1,74E-18	Glycerol-3-phosphate (4TMS)
219	28,599	3,67E-10	9,4357	9,81E-10	Alkane
220	132,54	1,95E-19	18,709	2,11E-18	NA
221	28,314	4,15E-10	9,3823	1,09E-09	Ornithine (3TMS)
222	5,5705	0,00161	2,7931	0,0021549	NA
224	25,819	1,27E-09	8,8961	3,16E-09	NA
225	5,8918	0,001139	2,9434	0,001542	NA
226	60,143	1,97E-14	13,706	9,74E-14	similar to Fructose Derivate
227	16,553	1,90E-07	6,7208	3,91E-07	NA
228	7,3738	0,000251	3,6008	0,0003585	similar to Fructose Derivate
229	6,0219	0,000992	3,0034	0,0013482	NA (Sugar)
234	10,727	1,25E-05	4,903	2,05E-05	NA
235	125,47	4,45E-19	18,351	4,31E-18	NA
237	36,593	1,63E-11	10,789	5,17E-11	NA (Sugar)
238	64,47	7,41E-15	14,13	3,82E-14	Glyceric acid-3-phosphate (4TMS)
239	108,58	3,85E-18	17,415	3,11E-17	Citric acid (4TMS)
240	384,3	1,49E-26	25,826	1,33E-24	NA
242	17,256	1,23E-07	6,9116	2,57E-07	NA
243	10,807	1,17E-05	4,9312	1,93E-05	NA
244	33,076	5,95E-11	10,225	1,81E-10	Galactose_1_5TMS
245	30,392	1,73E-10	9,7627	4,84E-10	NA
246	28,516	3,80E-10	9,4201	1,01E-09	Galactose_1_5TMS
247	4,4744	0,005517	2,2583	0,0070907	Altrose (1MEOX) (5TMS) BP
248	3,8933	0,010956	1,9604	0,013733	Psicose (1MEOX) (5TMS) BP
249	3,1627	0,026798	1,5719	0,03245	Allose (1MEOX) (5TMS) MP
251	17,317	1,18E-07	6,9279	2,50E-07	Sorbose (1MEOX) (5TMS) MP
252	14,916	5,56E-07	6,2553	1,08E-06	Fructose (1MEOX) (5TMS) MP
253	8,8132	6,48E-05	4,1885	9,99E-05	Ribose (1MEOX) (4TMS) BP
254	10,273	1,82E-05	4,7396	2,91E-05	Ribose (1MEOX) (4TMS) BP
255	10,883	1,10E-05	4,9583	1,84E-05	NA
257	11,534	6,54E-06	5,1842	1,13E-05	NA
258	24,888	1,97E-09	8,7056	4,87E-09	NA
261	6,763	0,000461	3,3367	0,0006429	NA (Sugar)
266	17,809	8,75E-08	7,0582	1,86E-07	NA (Sugar)
268	26,262	1,04E-09	8,9849	2,67E-09	NA (Sugar)
271	5,1242	0,002633	2,5796	0,0034843	Tyrosine (2TMS)
274	10,604	1,38E-05	4,859	2,25E-05	Galactose (1MEOX) (5TMS) MP

275	15,537	3,67E-07	6,4355	7,30E-07	Mannitol (6TMS)
281	3,745	0,013099	1,8828	0,016304	Alkane
282	20,574	1,78E-08	7,7484	4,05E-08	NA
284	3,4897	0,01788	1,7476	0,021949	NA
288	8,4924	8,68E-05	4,0615	0,0001315	Pentadecanoic acid, 14-methyl-, meth
291	201,53	3,34E-22	21,476	5,95E-21	NA
294	4,8448	0,003606	2,4429	0,0046882	NA426001
295	15,036	5,12E-07	6,2906	1,01E-06	NA426001
296	10,87	1,11E-05	4,9535	1,85E-05	Glucopyranose [-H20] (4TMS)
298	78,78	4,22E-16	15,374	2,64E-15	NA
299	11,466	6,90E-06	5,1609	1,19E-05	Glucopyranose, D- (5TMS)
300	13,167	1,90E-06	5,7214	3,47E-06	NA
302	4,8443	0,003608	2,4427	0,0046882	Galactose_1_5TMS
303	3,07	0,030091	1,5216	0,036068	Galactose_1_5TMS
305	6,1906	0,000831	3,0806	0,0011462	Gluconic acid-1,4-lactone (4TMS)
306	3,7592	0,012876	1,8902	0,016083	NA (Sugar)
311	12,777	2,53E-06	5,5969	4,55E-06	NA
312	6,8522	0,000421	3,3759	0,0005922	similar to NA (Inositol like)
314	355,21	5,11E-26	25,292	3,64E-24	NA
316	109,09	3,59E-18	17,445	2,97E-17	Hexadecenoic-acid_2_1TMS
319	45,613	8,83E-13	12,054	3,31E-12	NA
320	34,249	3,82E-11	10,418	1,17E-10	Hexadecanoic acid (1TMS)
321	13,211	1,84E-06	5,7356	3,37E-06	NA
322	125,42	4,48E-19	18,349	4,31E-18	Inositol, myo- (6TMS)
323	166,34	6,29E-21	20,202	8,29E-20	Xylulose-5-phosphate (1MEOX) (5TMS
324	256,99	7,87E-24	23,104	2,80E-22	NA (Sugar)
325	8,2345	0,00011	3,9578	0,0001648	NA
326	10,599	1,39E-05	4,8573	2,25E-05	NA (Sugar)
327	7,8905	0,000152	3,8172	0,0002232	NA (Sugar)
328	4,3681	0,006244	2,2045	0,0079959	NA
329	81,033	2,81E-16	15,551	1,79E-15	NA
330	52,65	1,25E-13	12,903	5,18E-13	Octadecadienoic acid methyl ester, 9,
331	203,08	2,97E-22	21,527	5,57E-21	NA
332	29,913	2,10E-10	9,6769	5,76E-10	NA
333	15,898	2,90E-07	6,5382	5,86E-07	NA
335	48,882	3,46E-13	12,461	1,37E-12	NA
336	28,995	3,10E-10	9,5093	8,41E-10	Octadecatrienoic acid methylester, 9,
337	241,14	2,11E-23	22,677	6,67E-22	NA
338	55,261	6,41E-14	13,193	2,85E-13	Octadecanoic acid methyl ester (FAM
340	10,656	1,33E-05	4,8776	2,16E-05	Heptadecanoic acid (1TMS)
341	8,7032	7,16E-05	4,1452	0,0001089	NA
343	45,784	8,39E-13	12,076	3,18E-12	NA
344	9,8076	2,70E-05	4,5685	4,29E-05	NA
345	43,793	1,53E-12	11,817	5,38E-12	Glycerophosphoglycerol (5TMS)
346	105,55	5,85E-18	17,233	4,63E-17	NA
347	85,883	1,21E-16	15,918	7,82E-16	NA
348	19,373	3,49E-08	7,457	7,67E-08	NA

349	30,228	1,85E-10	9,7335	5,14E-10	NA
350	58,853	2,67E-14	13,574	1,30E-13	Gluconic acid (6TMS)
351	170,47	4,33E-21	20,364	6,16E-20	Mannitol (6TMS)
352	101,91	9,83E-18	17,007	7,61E-17	NA
353	15,538	3,67E-07	6,4359	7,30E-07	NA
355	3,6706	0,014336	1,8436	0,017721	Ornithine, N2-acetyl- (4TMS)
358	3,4563	0,018628	1,7298	0,022789	Glycerol-3-phosphate (4TMS)
359	48,388	3,97E-13	12,401	1,55E-12	Octadecadienoic acid, n- (1TMS)
360	26,157	1,09E-09	8,9639	2,78E-09	Octadecenoic acid, 9-(E)- (1TMS)
361	98,053	1,74E-17	16,76	1,29E-16	NA
364	44,994	1,06E-12	11,974	3,93E-12	NA
365	54,138	8,51E-14	13,07	3,61E-13	NA
366	2,8067	0,041938	1,3774	0,049766	NA
368	14,125	9,57E-07	6,0189	1,80E-06	NA
369	70,835	1,94E-15	14,711	1,12E-14	Octadecanoic acid (1TMS)
371	42,257	2,46E-12	11,61	8,41E-12	NA
372	153,96	2,03E-20	19,691	2,50E-19	NA
373	28,735	3,46E-10	9,4611	9,33E-10	NA
374	7,9962	0,000138	3,8607	0,0002044	Mannose-6-phosphate (1MEOX) (6TM
375	7,1224	0,000321	3,4933	0,0004573	Xylulose-5-phosphate (1MEOX) (5TMS
377	5,8743	0,001161	2,9353	0,0015652	NA (Sugar-phosphate)
379	24,11	2,87E-09	8,5424	6,95E-09	NA
380	24,208	2,73E-09	8,5632	6,67E-09	NA (Sugar-phosphate)
381	6,6492	0,000517	3,2865	0,000719	NA (Sugar-phosphate)
382	54,48	7,80E-14	13,108	3,37E-13	NA
383	76,532	6,41E-16	15,193	3,87E-15	NA
384	15,633	3,44E-07	6,4631	6,92E-07	NA
385	76,835	6,06E-16	15,218	3,72E-15	NA
386	240,02	2,26E-23	22,645	6,67E-22	NA
387	86,769	1,04E-16	15,983	6,86E-16	NA
389	445,56	1,47E-27	26,834	1,74E-25	NA
390	223,46	6,82E-23	22,166	1,62E-21	NA
391	14,683	6,51E-07	6,1865	1,25E-06	Inositol-2-phosphate, myo- (7TMS)
392	11,264	8,11E-06	5,0912	1,37E-05	NA
393	61,42	1,47E-14	13,834	7,35E-14	NA
396	10,588	1,40E-05	4,8534	2,26E-05	NA
397	4,5804	0,004881	2,3115	0,0063181	NA
398	14,943	5,45E-07	6,2633	1,07E-06	NA
400	30,645	1,56E-10	9,8077	4,40E-10	NA
402	12,166	4,01E-06	5,3969	7,03E-06	NA
404	9,4016	3,84E-05	4,4157	5,98E-05	NA
406	92,474	4,10E-17	16,387	2,92E-16	NA
408	36,495	1,68E-11	10,774	5,31E-11	NA
414	12,396	3,37E-06	5,4728	5,96E-06	NA
415	16,852	1,58E-07	6,8026	3,28E-07	NA
416	126,11	4,13E-19	18,384	4,20E-18	NA (Sugar)
417	13,359	1,65E-06	5,7821	3,06E-06	NA

418	9,3991	3,85E-05	4,4147	5,98E-05	NA
419	13,248	1,79E-06	5,7472	3,30E-06	NA
420	19,703	2,90E-08	7,5382	6,44E-08	NA
422	11,395	7,30E-06	5,1366	1,25E-05	Kestose, 6- (11TMS)
424	26,813	8,06E-10	9,0939	2,09E-09	NA
425	11,378	7,40E-06	5,1307	1,26E-05	NA
426	37,027	1,40E-11	10,855	4,48E-11	NA
427	30,717	1,51E-10	9,8204	4,31E-10	Adenosine (3TMS) (Derivate not foun[illegible]
433	29,947	2,07E-10	9,683	5,73E-10	NA
434	23,193	4,52E-09	8,345	1,08E-08	NA
438	31,971	9,15E-11	10,039	2,74E-10	NA
439	31,109	1,29E-10	9,8894	3,76E-10	NA
441	15,527	3,69E-07	6,4327	7,30E-07	NA
442	10,569	1,42E-05	4,8467	2,28E-05	NA
447	20,071	2,36E-08	7,6278	5,27E-08	NA
451	40,692	4,05E-12	11,392	1,36E-11	NA
453	12,3	3,62E-06	5,4414	6,38E-06	NA
454	3,1541	0,027086	1,5673	0,032687	NA
455	3,5824	0,015962	1,7969	0,019663	NA

12-(Z,Z)-, n-

12,15-(Z,Z,Z)-, n-
